# PLCγ1 in dopamine neurons critically regulates striatal dopamine release via VMAT2 and synapsin III

**DOI:** 10.1038/s12276-023-01104-y

**Published:** 2023-11-01

**Authors:** Hye Yun Kim, Jieun Lee, Hyun-Jin Kim, Byeong Eun Lee, Jaewook Jeong, Eun Jeong Cho, Hyun-Jun Jang, Kyeong Jin Shin, Min Ji Kim, Young Chan Chae, Seung Eun Lee, Kyungjae Myung, Ja-Hyun Baik, Pann-Ghill Suh, Jae-Ick Kim

**Affiliations:** 1 Department of Biological Sciences, Ulsan National Institute of Science and Technology (UNIST), Ulsan, 44919 Republic of Korea; 2 Herbal Medicine Resources Research Center, Korea Institute of Oriental Medicine, Naju, 58245 Republic of Korea; 3 Department of Life Sciences, Korea University, Seoul, 02841 Korea; 4 Research Animal Resource Center, Korea Institute of Science and Technology (KIST), Seoul, 02792 Republic of Korea; 5 Center for Genomic Integrity, Institute for Basic Science (IBS), Ulsan, 44919 Republic of Korea; 6 Department of Biomedical Engineering, Ulsan National Institute of Science and Technology (UNIST), Ulsan, 44919 Republic of Korea; 7 Korea Brain Research Institute (KBRI), Daegu, 41062 Republic of Korea

**Keywords:** Cellular neuroscience, Neuronal physiology

## Abstract

Dopamine neurons are essential for voluntary movement, reward learning, and motivation, and their dysfunction is closely linked to various psychological and neurodegenerative diseases. Hence, understanding the detailed signaling mechanisms that functionally modulate dopamine neurons is crucial for the development of better therapeutic strategies against dopamine-related disorders. Phospholipase Cγ1 (PLCγ1) is a key enzyme in intracellular signaling that regulates diverse neuronal functions in the brain. It was proposed that PLCγ1 is implicated in the development of dopaminergic neurons, while the physiological function of PLCγ1 remains to be determined. In this study, we investigated the physiological role of PLCγ1, one of the key effector enzymes in intracellular signaling, in regulating dopaminergic function in vivo. We found that cell type-specific deletion of PLCγ1 does not adversely affect the development and cellular morphology of midbrain dopamine neurons but does facilitate dopamine release from dopaminergic axon terminals in the striatum. The enhancement of dopamine release was accompanied by increased colocalization of vesicular monoamine transporter 2 (VMAT2) at dopaminergic axon terminals. Notably, dopamine neuron-specific knockout of PLCγ1 also led to heightened expression and colocalization of synapsin III, which controls the trafficking of synaptic vesicles. Furthermore, the knockdown of VMAT2 and synapsin III in dopamine neurons resulted in a significant attenuation of dopamine release, while this attenuation was less severe in PLCγ1 cKO mice. Our findings suggest that PLCγ1 in dopamine neurons could critically modulate dopamine release at axon terminals by directly or indirectly interacting with synaptic machinery, including VMAT2 and synapsin III.

## PLCγ1: A Game Changer in Dopamine Release Mechanisms

Dopamine release is significantly increased in mice lacking the phospholipase Cγ1 (PLCγ1) enzyme in their dopamine neurons, according to a study by researchers at the Ulsan National Institute of Science and Technology. The team found that PLCγ1 conditional knockout mice exhibited normal dopamine neuron development and cellular structures, but showed enhanced dopamine release. This was potentially due to increased localization of vesicular monoamine transporter 2 (VMAT2) and synapsin III in dopaminergic axons. The findings suggest that PLCγ1 plays a critical role in regulating dopamine release by potentially modulating synaptic vesicle trafficking in dopamine neurons.

## Introduction

The monoamine neurotransmitter dopamine regulates various cognitive and psychomotor functions, including voluntary movement, reward-related learning, emotion, and motivation^1,2^. Dopamine is largely synthesized from dopamine neurons in the midbrain and released into the synaptic cleft, binding to dopamine receptors and activating dopamine receptor-mediated signaling in both presynaptic terminals and postsynaptic neurons^3,4^. Although a small set of neurons constitute dopamine neurons primarily in the substantia nigra pars compacta (SNc) and the ventral tegmental area (VTA) in the rodent and human midbrain^5^, they exert powerful modulatory effects on synaptic transmission and neuronal activity through extensive axonal arborization with numerous synaptic boutons^6,7^. These dopaminergic axons innervate diverse brain regions, such as the striatum, globus pallidus, cortex, and limbic areas, via nigrostriatal and mesocorticolimbic pathways^8^. Due to these structural and functional attributes, dysfunction of dopamine neurons can lead to severe neurological and psychiatric illnesses, including Parkinson’s disease^9,10^, schizophrenia^11,12^, attention deficit hyperactivity disorder (ADHD)^13^, depression^14^, and addiction^15^. In particular, selective degeneration of dopamine neurons in the SNc and their axon terminals decreases dopamine levels in the basal ganglia, which causes the main symptoms of Parkinson’s disease, such as tremor, muscle stiffness, and bradykinesia^9,10^. Many previous studies on dopamine and dopamine neurons have centered on the presynaptic and postsynaptic effects of dopamine through dopamine receptors and their downstream signaling^3,4^. However, there is a critically unmet need to better understand the intracellular signaling and molecular events by which dopamine neurons and their normal physiology are maintained.

Several intracellular signaling pathways have been implicated in the development and function of dopamine neurons. Among them, phospholipase C (PLC) is one of the most important intracellular signaling molecules in a variety of biological processes^16–20^. PLC is generally activated by extracellular signal-mediated receptors, and it hydrolyzes phosphatidylinositol 4,5-bisphosphate (PIP_2_) to generate diacylglycerol (DAG) and inositol 1,4,5-trisphosphate (IP_3_). These second messengers in turn regulate key cellular functions, including proliferation, differentiation, migration, and survival^21–25^. Mammalian PLC comprises 13 isozymes that are differentially expressed in bodily organs. Among them, PLCγ1, which is activated by receptor tyrosine kinase (RTK), is ubiquitously distributed in several organs and abundantly expressed in embryonal cortical structures, neurons, oligodendrocytes, and astrocytes in the brain^16^. PLCγ1 has been known to mediate pivotal neuronal functions in the brain, including neuronal development, synaptic transmission, and synaptic plasticity^26–28^. Interestingly, accumulating evidence indicates that PLCγ1 appears to modulate the functions of dopamine neurons. Chronic treatment with morphine, one of the opiates facilitating dopamine release, significantly increased PLCγ1 expression and phosphorylation in the ventral midbrain^29,30^. In addition, VTA region-specific overexpression of PLCγ1 was shown to regulate mood-related behaviors in rodents, such as sucrose preference, forced swimming, nociceptive stimuli, and elevated-plus maze behavior^31^. Despite these findings, our understanding of the physiological role and significance of PLCγ1 in dopamine neurons is far from complete, and the cell type-specific function of PLCγ1 in vivo remains unknown in dopamine neurons.

In this study, we focused on the physiological function of PLCγ1 in dopamine neurons in vivo by utilizing dopamine neuron-specific PLCγ1 conditional knockout mice. We found that genetic deletion of PLCγ1 does not negatively affect the development and cellular structures of dopamine neurons. Interestingly, while intrinsic properties were unaffected, the acute and chronic disruption of PLCγ1 in dopamine neurons markedly facilitated dopamine release, potentially through indirect interaction with VMAT2 and synapsin III. Furthermore, the possible role of VMAT2 and synapsin III in this enhanced dopamine release was confirmed by the knockdown of VMAT2 and synapsin III in dopamine neurons. Thus, our findings demonstrate that PLCγ1 critically affects dopamine release by potentially regulating synaptic vesicle trafficking in dopamine neurons.

## Materials and methods

### Animals

*Plcg1*^*F/F*^ mice (originally 129XC57BL/6 background, backcrossed with C57BL/6J mice for at least eight generations) were generated as previously described^26,27^. To produce dopamine neuron-specific PLCγ1 conditional knockout mice, *DAT-Cre* mice (B6.SJL-*Slc6a3*^*tm1.1(cre)Bkmn*^*/*J, Jackson stock No. 006660) were crossed with *Plcg1*^*F/F*^ mice, generating *DAT-Cre;Plcg1*^*F/F*^ mice. *DAT-Cre* mice were used as the control group. In optogenetic experiments, *DAT-Cre;Plcg1*^*F/F*^ mice were crossed with Ai32 mice (B6.Cg-*Gt(ROSA)26Sor*^tm32(CAG-COP4*H134R/EYFP)Hze^/J, Jackson stock No. 024109) to selectively express channelrhodopsin-2 in dopamine neurons, producing *DAT-Cre;Ai32*;*Plcg1*^*F/F*^ mice. *DAT-Cre;Ai32* mice were used as the control group in optogenetic experiments. Adult (2–5 months old) male and female mice were used in most of the experiments. Young (3–4 weeks old) male and female mice were used for whole-cell patch clamp recording due to the difficulty in making healthy whole-cell configurations from midbrain dopamine neurons in adult mice. Mice used for this study were maintained under consistent housing conditions on a 12-h light/dark cycle (lights on from 6 a.m. to 6 p.m.) in groups of up to 5 mice per cage. Food and water were available *ad libitum*. The mice were bred under standard pathogen-free housing conditions in the animal facility of the Ulsan National Institute of Science and Technology (UNIST). The experimenter was not blind to the genotypes of the mice. All experimental procedures were conducted in accordance with protocols approved by the Institutional Animal Care and Utilization Committee of UNIST (UNISTIACUC-20-18).

### Western blot

Mice were anesthetized with isoflurane inhalation (TERRELL™ Isoflurane, Piramal Critical Care Inc., USA) and decapitated. Brains were immediately dissected in 4 °C cold ACSF solution. Coronal striatum and midbrain slices were prepared at 1000 μm thickness using a vibratome (VT1200, Leica). Using dissection tools, we specifically isolated the dorsal striatum from coronal striatal slices. Proteins were extracted from brain slices using RIPA Lysis and Extraction Buffer (Thermo Scientific, 89900). Halt™ Protease and Phosphatase Inhibitor Cocktail (Thermo Scientific, 78440) was also freshly added. A Pierce™ BCA Protein Assay Kit (Thermo Scientific, 23227) was used to measure the protein concentration. Proteins with 5X SDS‒PAGE loading buffer (iNtRON, IBS-BS024) were denatured at 95 °C for 5 min. Protein samples were loaded in the gel, electrophoresed, and transferred to a 0.45 μm nitrocellulose membrane (Cytiva, 10600096). The membranes were blocked with 5% skim milk in TTBS and incubated with primary antibodies at 4 °C overnight. Primary antibodies used for western blotting targeted PLCγ1 (generated in the lab, 1:1000)^26,27,32^, TH (Abcam, ab112, 1:1000), DAT (Abcam, ab5990, 1:1000), synapsin I (Synaptic systems, 106 011, 1:1000), VMAT2 (Frontier institute, VMAT2-Rb-Af720, 1:1000), synapsin II (Synaptic systems, 106 203, 1:1000), synapsin III (Synaptic systems, 106 304, 1:1000), β-actin (GeneTex, GTX629630, 1:5000), and GAPDH (Invitrogen, AM4300, 1:1000). After washing, membranes were incubated with secondary HRP-conjugated antibodies (VWR, 1:5000) for 1 h at room temperature. After washing three times, protein bands were visualized by Clarity Western ECL Substrate (Bio-Rad, 1705061) with an Amersham Imager (Cytiva, 29270769). Protein bands were quantified by ImageJ software. Before reprobing, membranes were stripped for 30 min at 65 °C in a stripping buffer (iNtRON, IBS-BS018). After washing three times, the membranes were blocked and sequentially reprobed with antibodies.

### Preparation of synaptosomal fraction

The synaptosomal fraction was prepared as previously described^26,33^. Coronal striatum slices were homogenized in lysis buffer (0.32 M sucrose, 4 mM HEPES, 2 mM EDTA, protease, and phosphatase inhibitor cocktail). The homogenates were centrifuged at 1000×*g* for 10 min at 4 °C. The supernatants were collected and centrifuged at 12,500×*g* for 20 min at 4 °C. The supernatants containing the cytosolic and light membrane fractions were discarded, and the pellets containing the crude synaptosomal fraction were resuspended in lysis buffer for western blotting.

### Immunohistochemistry

Mice were anesthetized with a zoletile (60 mg/kg, Virbac Korea) and rompun (15 mg/kg, Bayer Korea) mixture solution and transcardially perfused with 0.1 M PB, followed by 4% paraformaldehyde (PFA) in 0.1 M PB. The dissected brains were further fixed in 4% PFA overnight, followed by cryoprotection in 30% sucrose in 0.01 M PB overnight at 4 °C. For the staining of PLCγ1, brains were fixed in 4% PFA for 4 h. Sections (40 μm thickness) were coronally cut using a microtome (Leica, SM2010). Free-floating sections were washed in PBS for 10 min and incubated in 0.5% PBST (0.5% Triton X-100 in PBS) for 10 min at room temperature (RT). Sections were blocked in 2% bovine serum albumin and 10% normal goat serum in PBST for 1 h at RT and incubated with primary antibodies diluted in a blocking solution overnight at 4 °C. Following three washes in PBST, the sections were incubated with Alexa Fluor-tagged secondary antibodies at a 1:1000 dilution for 2 h at room temperature. Primary antibodies used in this study targeted PLCγ1 (generated in the lab, 1:500)^26,27,32^, TH (Abcam, ab112, 1:1000; Abcam, ab76442, 1:500), DAT (Abcam, ab5990, 1:1000), VMAT2 (Frontier Institute, VMAT2-Rb-Af720, 1:200), D2R (Frontier Institute, D2R-GP-Af500, 1:100), synapsin I (Synaptic Systems, 106 011, 1:1000), synapsin II (Synaptic Systems, 106 203, 1:200), synapsin III (Synaptic Systems, 106 304, 1:500), bassoon (Synaptic Systems, 141 004, 1:500), GABA_A_ receptor α1 subunit (Frontier Institute, GABAARa1-Rb-Af660, 1:200), VGAT (Synaptic Systems, 131 004, 1:500), Gephyrin (Synaptic Systems, 147 111, 1:500), and PIP_2_ (Santa Cruz, sc-53412). The secondary antibodies were Alexa Fluor 405-, 488-, 594-, and 647-conjugated antibodies (Thermo Scientific). After washing with PBST and PBS, sections were mounted with ProLong™ Gold Antifade Mountant (Invitrogen, P36934). For quantitative analysis, images were captured by using an FV1000 confocal laser scanning microscope (Olympus) and an LSM 880 confocal laser scanning microscope with Airyscan (Carl Zeiss). To quantify the dopamine neurons in the SNc and VTA, an FV1000 confocal microscope with a ×20 or ×63 lens was used. The images were analyzed using ImageJ software (NIH) to quantify the fluorescence-positive area and mean intensity. Confocal 3D images were acquired using the z-stack function of an LSM880 confocal laser scanning microscope with Airyscan (Carl Zeiss). Images were collected at 2 μm intervals with a ×63 oil-immersion objective, ×3 optical zoom, and a resolution of 1248 × 1248 pixels. 3D images were reconstructed using IMARIS 9.6 software. Acquired images were further analyzed by Zen software (Carl Zeiss), ImageJ program (NIH, measure function), and MATLAB (MathWorks, custom codes). For DAB (3,3’-diaminobenzidine) staining, brain slices were mounted on slides and allowed to dry overnight at RT. The slides were washed in PBS for 10 min, followed by incubation in 0.3% H_2_O_2_ in PBS for 10 min to block endogenous peroxidase activity. After two additional PBS washes, the slides were permeabilized in 0.5% PBST for 10 min and subsequently blocked with a blocking solution for 1 h. Next, the slides were incubated with TH antibody (Abcam, ab112, 1:1000) in blocking solution at 4 °C overnight. Following rinsing, the slides were incubated with HRP-conjugated secondary antibody (VWR, 1:1000) for 2 h at RT. After rinsing in PBS, the sections were exposed to DAB solution (Vector Labs, SK-4100) for 2 min, followed by dehydration in a graded series of ethanol (70–100%) for 30 sec and allowed to air dry. Finally, cover slips were applied using a permanent mounting medium (VectaMount, H-5000). For quantitative analysis, images were captured using a virtual microscope (Olympus).

### Counting dopamine neurons and 2D-based stereological analysis

The number of TH-positive fluorescent cells was quantified using the ImageJ cell count plugin. We counted the TH-positive dopamine neurons co-labeled by DAPI. The number of dopamine neurons in the midbrain dramatically varies depending on the rostral-to-caudal location of the coronal slices. In our data, we chose a single slice from each coronal location (rostral, intermediate, caudal), counted the number of dopamine neurons from each slice, and then summed these numbers (we separated left and right hemispheres, so we obtained a total of six slices from one mouse). For stereological counting of dopamine neurons, the stereological analysis method was used as described previously^34,35^. The images were captured by using a THUNDER Imager Live Cell (Leica). Images were collected with a ×20 lens and a resolution of 1248 × 1248 pixels.

### Laser capture microdissection

Mice were anesthetized with isoflurane inhalation (TERRELL™ Isoflurane, Piramal Critical Care Inc., USA) and decapitated. The brain was then extracted and embedded in OCT compound, followed by freezing on dry ice and storage at −80 °C. Slicing was performed using a cryostat at a thickness of 20 μm, and the sections were mounted on PEN membrane slides (Carl Zeiss™, 15350731). For TH antibody staining, we referred to a previous study^36^. Briefly, the tissue was fixed in a solution of acetone–methanol (1:1) at −20 °C for 10 min. The slides were rinsed in RNase-free 1% PBST. The sections were covered with 200 μl of PBST containing tyrosine hydroxylase antibody (Abcam, ab112, diluted 1:100), along with 400 U/ml of RNaseOUT™ Recombinant Ribonuclease Inhibitor (Invitrogen™, 10777019). After a 15 min incubation, the slides were briefly rinsed in PBS. Next, the tissue was covered with 200 μl of HRP-conjugated secondary antibody diluted 1:100 in PBST with 400 U/ml ribonuclease inhibitor and incubated for 10 min. After rinsing in PBS, the sections were treated with DAB solution (Vector Labs, SK-4100) for 2 min, followed by dehydration in a graded series of RNase-free ethanol (70–100%) for 30 s and allowed to air dry. Using the PALM MicroBeam laser microdissection system (Carl Zeiss), more than 1000 TH-positive dopamine neurons were collected from a single mouse.

### RNA extraction and quantitative real-time PCR

Isolated dopamine neurons were used for total RNA extraction using the PicoPure™ RNA Isolation Kit (Thermo Scientific, KIT0204, following the manufacturer’s instructions). Reverse transcription of the extracted RNA was carried out using the High-Capacity RNA-to-cDNA Kit (Thermo Fisher, 4387406). Two hundred nanograms of total RNA was used as input for cDNA synthesis. Quantitative real-time PCR was performed using the LightCycler 480 II Real-Time PCR system (Roche) and Prime Q-Mastermix (Genet Bio, Q-9200). Specific primers for each target mRNA were used in the qPCR. The expression level of each mRNA was normalized to that of GAPDH, which was used as the internal control. We used the following primers: PLCβ1 (forward 5′-CTGCG AGAAA GAGAA GAAGG-3′, reverse 5′-TACTG AACCA CCTCC TGGAT-3′), PLCβ2 (forward 5′-CGACG AGATC TTCAC TTCCT-3′, reverse 5′-TGGGT TCGTA CTTGT CAATG-3′), PLCβ3 (forward 5′- TGCCT GCCCT GCTTA TCTAC A-3′, reverse 5′-TAGGC TTACG TGCTT GATGG G-3′), PLCβ4 (forward 5′-CCACC GACAC CATAC GGAAA-3′, reverse 5′-GGAGA TGTGT CGGTA GCCT-3′), PLCγ1 (forward 5′-GGGAC TTTGA CCGCT ACCAA-3′, reverse 5′-CCGGA GCCAC CTCTC AATTT-3′), and PLCγ2 (forward 5′-CATGA ACAGC AGGAG CTATG-3′, reverse 5′-GATCC AGTAG TGGGA CAAGG-3′).

### Expansion microscopy imaging

For immunofluorescence staining, brain slices were stained following the immunohistochemistry protocol described above until they were ready to mount to slide glasses. Expansion of brain slices was performed as previously described^37^. Brain slices were maintained in the AcX solution overnight. After washing with PBS, brain slices were incubated in a gelling solution for 30 min at 4 °C. These slices were then mounted onto the gelation chamber and incubated for 2 h at 37 °C. The gels were then trimmed and submerged in a digestion buffer overnight. Finally, the gels were expanded in water for 20 min three times and were mounted onto coverslips coated with poly-l-lysine for imaging. For quantitative analysis, images were captured by using an LSM 980 confocal laser scanning microscope (Carl Zeiss). Images were collected with a ×40 water-immersion objective, 2 × 2 stitched images, and a resolution of 4894 × 4894 pixels. The expansion factor of the images was measured as 5. Acquired images were analyzed using Zen software (Carl Zeiss), ImageJ (NIH), and MATLAB (MathWorks, custom codes).

### Brain slice preparation for electrophysiology

Brain slices were prepared as previously described^38^. Coronal brain slices containing the DStr-NAc (300 μm thick) or SNc-VTA (250 μm thick) were obtained for whole-cell patch clamp recording and fast-scanning cyclic voltammetry (FSCV). Mice were anesthetized with isoflurane (Piramal Critical Care), decapitated, and the brain was briefly exposed to ice-chilled artificial cerebrospinal fluid (ACSF) containing 125 mM NaCl, 2.5 mM KCl, 1.25 mM NaH_2_PO_4_, 25 mM NaHCO_3_, 1 mM MgCl_2_, 2 mM CaCl_2_ and 15 mM glucose oxygenated with 95% O_2_ and 5% CO_2_. Acute brain slices were prepared using a tissue vibratome (Leica, VT 1200S) in ice-cold ACSF. Brain slices were first maintained in ACSF for 30 min at 34 °C and then another 30 min at room temperature. After recovery, slices were transferred to a submerged recording chamber perfused with ACSF. The temperature of the ACSF was maintained at 30–31 °C by an in-line solution heater (Warner Instruments) with a flow rate of 2–3 ml/min. Brain slices were used for electrophysiological recording within 5 h after recovery.

### Electrophysiology

Electrophysiology was performed as previously described^38^. Dopamine neurons were visualized using a conventional infrared (IR)-differential interference contrast (DIC) microscope (BX51WI, Olympus). Whole-cell voltage-clamp recordings were performed using borosilicate glass pipettes (2.5–3.5 MΩ) filled with Cs^+^-based low Cl^−^ internal solution containing 135 mM CsMeSO_3_, 10 mM HEPES, 1 mM EGTA, 3.3 mM QX-314, 0.1 mM CaCl_2_, 4 mM Mg-ATP, 0.3 mM Na_3_-GTP, and 8 mM Na_2_-phosphocreatine (290–300 mOsm, pH 7.3 with CsOH). To measure inhibitory and excitatory synaptic currents from the same neuron, the membrane potential was first held at +0 mV (reversal potential of ionotropic glutamate receptors) to measure inhibitory synaptic currents and then continuously held at −70 mV (reversal potential of chloride) to measure excitatory synaptic currents under the voltage clamp mode (liquid junction potential corrected). The access resistance was 10–20 MΩ, and only cells with a change in access resistance <20% were included in the analysis. The signals were collected using Multiclamp 700B (Molecular Devices), low-pass filtered at 2 kHz, and digitized at 10 kHz (NI PCIe-6259, National Instruments). Recording data were monitored and acquired by WinWCP (Strathclyde software, http://spider.science.strath.ac.uk/sipbs/software_ses.htm) and further analyzed offline using Clampfit 10.7 software (Molecular Devices) and OriginPro 2017 (OriginLab). For mEPSC and mIPSC measurements, tetrodotoxin (500 nM, alomone labs, T-550) and D-AP5 (25 μM, Tocris, Cat. no. 0106) were included in ACSF. The amplitude and frequency of mEPSCs and mIPSCs were analyzed using Mini Analysis software (Synaptosoft); spontaneous events were first detected by Mini software, and then individual spontaneous events were manually checked again to increase the accuracy of the analysis. To examine the intrinsic properties of dopamine neurons, the potassium-based internal solution containing 135 mM KMeSO_3_, 3 mM KCl, 10 mM HEPES, 1 mM EGTA, 0.1 mM CaCl_2_, 8 mM Na_2_-phosphocreatine, 4 mM Mg-ATP, and 0.3 mM Na_3_-GTP (290–300 mOsm, pH 7.3 with KOH) was used under the current clamp mode. Spontaneous firing of dopamine neurons was measured for 1 min, and the average firing frequency was calculated. To measure input resistance and detect voltage sag from dopamine neurons, a stepwise hyperpolarizing current (−150 to 0, 50 pA step) was injected for 1 sec (interpulse interval, 20 sec), and the amplitude of voltage sag was measured. Voltage sag was determined as the difference between the instantaneous voltage (peak hyperpolarization) and steady-state voltage during 1 sec of current injection. To examine the membrane excitability of dopamine neurons, a stepwise depolarizing current (50–150, 50 pA step) was injected for 1 sec (interpulse interval, 20 s), and the total number of action potentials was counted. To induce hyperpolarization-activated current (Ih current) in dopamine neurons, voltage-clamp recording was carried out, and holding potentials were continuously held at different negative potentials (−70 to −120, 10 mV decrement) for 1 s. Ih is calculated as the current difference between the instantaneous current and steady-state inward current during 1 s. To stimulate ChR2-expressing dopaminergic axons, blue laser light (450 nm, 1 ms pulse with 60 s intervals, 50% saturation power under the objective <10 mW) from a diode laser (MDL-III-450, Opto Engine LLC) was focused on the back focal plane of the objective to generate wide-field illumination. GABA (IPSC) and glutamate (EPSC) cotransmission from DA axon terminals was recorded from the medium spiny neurons (MSNs) in the DStr by optogenetically activating DA terminals.

### Biocytin labeling and immunostaining of biocytin-filled neurons

Biocytin labeling was performed as previously described^38^. To examine the morphology, dendritic arborization, and number of dendritic spines in a single dopamine neuron, the potassium-based internal solution with 0.2% biocytin (Sigma-Aldrich, B4261) was loaded into dopamine neurons in the SNc and VTA for 30 min. After the delicate removal of the glass pipette, brain slices were fixed with 4% PFA overnight at 4 °C. On the following day, slices were washed with PBS, permeabilized with PBST, and blocked with PBST solution containing 10% normal goat serum and 2% bovine serum albumin for 1 h. Brain slices were then incubated with Alexa 594-conjugated streptavidin (Invitrogen, S-11227, 1:500) in a blocking solution for 2 h at room temperature. After washing with PBST and PBS, brain slices were mounted on slides using a mounting medium containing DAPI (Invitrogen, P36935). Images were obtained by using an FV1000 confocal laser scanning microscope (Olympus). Images of dendritic spines were acquired with a ×100 oil-immersion lens and ×2 optical zoom. We chose one secondary dendrite with the highest number of spines in each neuron for the analysis of spine density. We only included spine-like structures in our analysis to calculate spine densities. Sholl analysis was performed by using the ImageJ Sholl Analysis plugin.

### Fast-scan cyclic voltammetry (FSCV)

FSCV was performed as previously described^38^. Extracellular dopamine release was recorded by FSCV using carbon-fiber microelectrodes (7 μm diameter carbon fiber sealed by glass, 50–100 μm exposed tip length beyond the tapered glass seal) at the DStr and NAc core. To detect dopamine release, a triangular scanning waveform was applied to the carbon fiber electrode from −0.4 V (vs. AgCl reference electrode) to +1.3 V and back (8.5 ms waveform width) at a scan rate of 400 V/s (repeated at 10 Hz). The carbon fiber electrode was held at −0.4 V between scans. Cyclic voltammograms were background-subtracted by averaging 10 background scans. To evoke dopamine release by optogenetic stimulation, ChR2-expressing axon terminals of dopamine neurons were stimulated by blue laser light (450 nm, 2 ms single pulse width at 25 Hz for one pulse stimulation and five pulses stimulation). We used 50% saturation power under the objective (less than 10 mW, diode laser). For electrical stimulation, a bipolar concentric electrode (CBAPB75, FHC) was placed on the surface of the slice close to the carbon fiber electrode. Dopamine release was evoked by an electrical current (2 ms pulse duration for one pulse stimulation and five pulses stimulation) controlled by an ISO-flex stimulus isolator (A.M.P.I., Israel) or DS-3 stimulus isolator (Digitimer, UK). We used the current intensity by which 50% of maximal dopamine release could be induced. The oxidation current by optogenetically or electrically evoked dopamine release was detected and monitored using the TarHeel CV system. Changes in dopamine concentration were quantified by plotting the peak oxidation current of the voltammogram over time. The carbon fiber electrode was calibrated at the end of each experiment to convert the oxidation current to dopamine concentration using dopamine (Tocris, 3548, 10 μM) in ACSF. U73122 (Sigma, U6756, 10 μM, dissolved in DMSO), reserpine (Sigma, R0875, 3 μM), and quinpirole (Tocris, 1061, 50 nM) were used in the corresponding experiments.

### shRNA expression and verification of shRNA-mediated knockdown of VMAT2 and synapsin III

VMAT2, synapsin III-specific, and control shRNAs were expressed in the pSicoR vector (Addgene, 21907)^39^. We tested three different pSicoR vectors encoding VMAT2 or synapsin III-specific shRNA expression cassettes for their ability to knock down target gene expression in HEK293T (ATCC® CRL3216™) cells; the pSicoR-control served as a nontarget control shRNA containing nonhuman or mouse shRNA (5′-TCGCA TAGCG TATGC CGTT-3′). Three kinds of shRNA were synthesized by the following method: Two kinds of oligos were purchased (Sequence of oligos; [phos]5′-t [sense sequence of target] ttcaagaga [reverse complement sequence of target] ttttttc-3′ and [phos]5′-tcgagaaaaaa [sense sequence of target] tctcttgaa [reverse complement sequence of target] a-3′). The sequence information of the three candidates for the shRNA is as follows: mVMAT2-sh1 (5′-TATCT CATTG GAACC AATAT T-3′), mVMAT2-sh2 (5′-TCCTT GGCTC ATGAC AATTA T-3′), mVMAT2-sh3 (5′-ACAGC ATCTT CTCTT ACTAT A-3′), mSyn3-sh1 (5′-ACCCA GGATC TTGTT GGTTA T-3′), mSyn3-sh2 (5′- GAGCT GAACC TGGCT GCTTA T-3′), and mSyn3-sh3 (5′-TCCTT GGGTC CTGAG AAATT T-3′). The two oligos were annealed with annealing buffer (200 mM potassium acetate, 60 mM HEPES–KOH, 4 mM Mg-acetate, pH 7.3 was adjusted by KOH) and incubated at 95 °C for 5 min and 70 °C for 10 min. The annealed double-stranded oligo was inserted into HpaI-XhoI restriction enzyme sites within the pSicoR vector and verified by sequencing.

### Quantitative real-time polymerase chain reaction

Quantitative real-time polymerase chain reaction (qRT-PCR) assays for gene silencing that targeted VMAT2 and synapsin III candidates were used. All shRNAs were transfected into HEK293T cells. Approximately 24 h after transfection, total RNA was isolated from HEK293T cells using an RNA extraction kit (Intron, 17221) according to the manufacturer’s instructions. Complementary DNA (cDNA) was synthesized from 1 μg of total RNA, and reverse transcription was performed using a SuperScript cDNA synthesis kit (Bioneer, AccuPower® CycleScript™ RT PreMix & Master Mix, K-2044) according to the manufacturer’s instructions. qRT-PCR was performed using a SYBR Green expression assay (Thermo Fisher Scientific, Power SYBR® Green Master Mix, 4367659). Primer sets for target genes were purchased from Macrogen: VMAT2 (forward 5′-CCTCT TACGA CCTTG CTGAA GG-3′, reverse 5′-GCTGC CACTT TCGGG AACAC AT-3′) and synapsin III (forward 5′-TTATC GATGA CGCCC ATACA-3′, reverse 5′-AGCCT CCGGT AACAT AAGCA-3′). GAPDH is a housekeeping gene that was used as a control. The delta-delta Ct method was used to calculate fold changes in gene expression^40^. Among the three shRNA candidates, we selected the most effective shRNA candidate to achieve target gene knockdown for AAV production.

### AAV cloning and production

For adeno-associated virus (AAV)-based shRNA expression, pSicoR-shRNA vectors with XbaI/HindIII-containing enzymatic sites were inserted into the pAAV-minCMV-mCherry (Addgene, 27970) vector by the same enzymatic site. AAV virus was thereafter purified by iodixanol gradient ultracentrifugation by the KIST Virus Facility. The virus titers were as follows: AAV5-U6-scr-CMV-mCherry (1.1 × 10^14^ genome copies/ml), AAV5-U6-mVMAT2-sh3-CMV-mCherry (1.09 × 10^14^ genome copies/ml), and AAV5-U6-mSyn III-sh1-CMV-mCherry (1.02 × 10^14^ genome copies/ml).

### Stereotaxic 6-OHDA injection

Stereotaxic 6-OHDA injection was conducted on over 10–20-week-old male and female mice (*DAT-Cre*, *DAT-Cre;Plcg1*^*F/F*^) and performed using a stereotaxic system (51730, Stoelting). Before surgery, mice were deeply anesthetized by intraperitoneal injection of a zoletil (60 mg/kg, Virbac Korea) and rompun (15 mg/kg, Bayer Korea) mixture solution (zoletil:rompun:saline = 4:1:20). A total volume of 300 nl 6-OHDA solution (1.25 mg/ml, dissolved in 0.9% sterile saline with 0.02% ascorbic acid) was injected unilaterally into the left MFB (coordinates used, AP: −1.2 mm, ML: +1.2 mm from bregma, DV: −4.75 mm from exposed dura mater). A glass micropipette with a long narrow tip (size: 10–20 μm) was made using a micropipette puller (P-1000, Sutter Instrument) to deliver 6-OHDA. The glass pipette slowly reached the target area and was left for 5 min before 6-OHDA injection. The 6-OHDA solution was injected at an infusion rate of 100 nl/min and withdrawn 10 min after the end of the injection. After injection, the scalp was sutured, and the mice were returned to their home cages. 6-OHDA-injected mice were used for electrophysiology and immunohistochemistry experiments 3 days after injection.

### Stereotaxic Cre virus injection for acute knockout of PLCγ1 in adult mice

Stereotaxic virus injection was conducted on 8- to 12-week-old male and female mice (WT, *Plcg1*^*F/F*^) and performed using a stereotaxic system (51730, Stoelting). Before surgery, mice were deeply anesthetized by intraperitoneal injection of a zoletil (60 mg/kg, Virbac Korea) and rompun (15 mg/kg, Bayer Korea) mixture solution (zoletil:rompun:saline = 4:1:20). A total volume of 700 nl Cre virus solution (AAV8-hsyn-GFP-Cre, UNC vector core, AV5053C) was injected bilaterally into the SNc (coordinates used, AP: −3.1 mm, ML: ±1.2 mm from bregma, DV: −4.0 mm from exposed dura mater). The virus titer was 6.5 × 10^12^ virus molecules/ml for AAV8-hsyn-GFP-Cre. A glass micropipette with a long narrow tip (size: 10–20 μm) was made using a micropipette puller (P-1000, Sutter Instrument) to deliver the virus. The glass pipette slowly reached the target area and was left for 5 min before the virus injection. Virus solution was injected at an infusion rate of 100 nl/min and withdrawn 7 min after the end of injection. After injection, the scalp was sutured, and the mice were returned to their home cages. Virus-injected mice were used for electrophysiology and immunohistochemistry experiments 4 weeks after injection.

### Stereotaxic viral injection for VMAT2 and synapsin III knockdown

Stereotaxic virus injection was conducted on 6- to 8-week-old male and female mice (*DAT-Cre*, *DAT-Cre;Plcg1*^*F/F*^) and performed using a stereotaxic system (51730, Stoelting). Before surgery, mice were deeply anesthetized by intraperitoneal injection of a zoletil (60 mg/kg, Virbac Korea) and rompun (15 mg/kg, Bayer Korea) mixture solution (zoletil:rompun:saline = 4:1:20). A total volume of 1 μl of control virus solution (AAV5-U6-scr-CMV-mCherry for VMAT2 and synapsin III knockdown) was injected unilaterally into the left SNc (coordinates used, AP: -3.1 mm, ML: +1.2 mm from bregma, DV: −4.0 mm from exposed dura mater), and a total volume of 1 μl of KD virus solution (AAV5-U6-mVMAT2-sh3-CMV-mCherry, AAV5-U6-mSyn3-sh1-CMV-mCherry) was injected unilaterally into the right SNc (coordinates used, AP: −3.1 mm, ML: −1.2 mm from bregma, DV: −4.0 mm from exposed dura mater). A glass micropipette with a long narrow tip (size: 10–20 μm) was made using a micropipette puller (P-1000, Sutter Instrument) to deliver the virus. The glass pipette slowly reached the target area and was left for 5 min before the virus injection. Virus solution was injected at an infusion rate of 100 nl/min and the micropipette was withdrawn 10 min after the end of injection. After injection, the scalp was sutured, and the mice were returned to their home cages. Virus-injected mice were used for electrophysiology and immunohistochemistry experiments 4 weeks after injection.

### Statistics

We performed all statistical analyses using GraphPad Prism software (version 9.5, GraphPad Software). The appropriate statistical methods for each experiment are described in the figure legend. Summary graphs are all shown as the mean ± SEM. Unpaired Student’s *t* test and repeated measures two-way ANOVA with post hoc Sidak’s multiple comparison test were used to determine significant differences between the genotypes. A *P* value < 0.05 was considered statistically significant. *P < 0.05, **P < 0.01, ***P < 0.001, ****P < 0.0001.

## Results

### Cell type-specific deletion of PLCγ1 in dopamine neurons does not negatively affect cell survival or the expression of dopaminergic markers

**Fig. 1:**
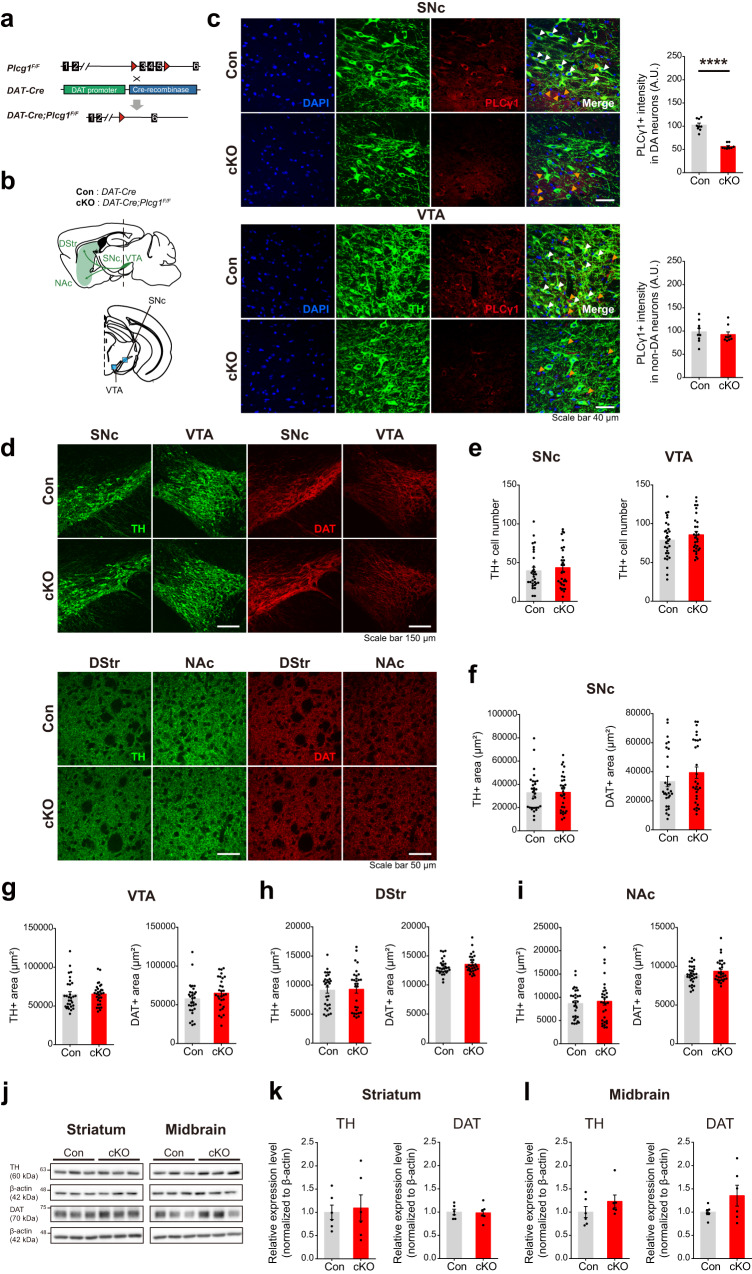
Cell type-specific deletion of PLCγ1 in dopamine neurons does not negatively affect cell survival or the expression of dopaminergic markers. **a** Breeding scheme for the generation of dopamine neuron-specific PLCγ1 conditional knockout mice. **b** Anatomical location of dopamine neurons in the SNc and VTA regions and their axonal projections. **c** Depletion of PLCγ1 in the dopamine neurons of the SNc and VTA in *DAT-Cre;Plcg1*^*F/F*^ mice and quantification of the mean intensity of PLCγ1 in dopamine neurons (white arrowhead, expression of PLCγ1 in dopamine neurons; orange arrowhead, expression of PLCγ1 in nondopamine neurons; unpaired two-tailed *t*-test, *n* = 9 images from three mice per genotype; PLCγ1+ intensity in dopamine neurons, Con 102.6 ± 4.171, cKO 56.59 ± 1.898, ****p < 0.0001; PLCγ1+ intensity in nondopamine neurons, Con 98.48 ± 7.829, cKO 92.56 ± 5.758, *p* = 0.5505). **d** Representative confocal images of TH and DAT expression in cell bodies (SNc and VTA) and axons (DStr and NAc) of dopamine neurons from control and PLCγ1 cKO mice. **e** The number of TH-positive cells in the SNc and VTA (unpaired two-tailed *t*-test, *n* = 30 images from five mice per genotype; SNc, Con 39.6 ± 4.41 cells, cKO 43.67 ± 4.828 cells, *p* = 0.5364; VTA, Con 78.73 ± 4.577 cells, cKO 85.8 ± 4.311 cells, *p* = 0.2657). **f**, **g** Quantification of TH-positive and DAT-positive areas in the SNc (**f**) and VTA (**g**) (unpaired two-tailed *t*-test, *n* = 30 images from five mice per genotype; SNc TH+ area, Con 32784 ± 2944 μm^2^, cKO 33153 ± 2843 μm^2^, *p* = 0.9286; SNc DAT+ area, Con 33236 ± 3573 μm^2^, cKO 39387 ± 3919 μm^2^, *p* = 0.2509; VTA TH+ area, Con 64909 ± 3560 μm^2^, cKO 65412 ± 2693 μm^2^, *p* = 0.9084; VTA DAT+ area, Con 57680 ± 3783 μm^2^, cKO 64986 ± 3677 μm^2^, *p* = 0.1714). **h**, **i** Quantification of TH-positive and DAT-positive axons of dopamine neurons in the DStr (**h**) and NAc (**i**) (unpaired two-tailed *t*-test, *n* = 30 images from five mice per genotype; DStr TH+ area, Con 9129 ± 526.2 μm^2^, cKO 9271 ± 658.6 μm^2^, *p* = 0.8670; DStr DAT+ area, Con 13062 ± 241.6 μm^2^, cKO 13553 ± 281.9 μm^2^, *p* = 0.1911; NAc TH+ area, Con 8650 ± 576.3 μm^2^, cKO 9128 ± 831 μm^2^, *p* = 0.6382; NAc DAT+ area, Con 8888 ± 210.2 μm^2^, cKO 9357 ± 261.2 μm^2^, *p* = 0.1673). **j** Representative western blot analysis of the expression of TH and DAT in the striatum and midbrain tissue from control and PLCγ1 cKO mice. **k**, **l** Quantification of TH (**k**) and DAT expression (**l**) in the striatum and midbrain from western blot analysis (unpaired two-tailed *t*-test, *n* = 6 mice per genotype; striatum TH, Con 1 ± 0.056, cKO 1.352 ± 0.225, *p* = 0.1598; striatum DAT, Con 1 ± 0.06, cKO 0.982 ± 0.073, *p* = 0.8592; midbrain TH, Con 1 ± 0.12, cKO 1.229 ± 0.139, *p* = 0.2394; midbrain DAT, Con 1 ± 0.154, cKO 1.094 ± 0.281, *p* = 0.7752).

To unravel the physiological roles of PLCγ1 in dopamine neurons in vivo, we generated dopamine neuron-specific PLCγ1 knockout mice (*DAT-IRES-Cre;Plcg1*^*F/F*^, PLCγ1 cKO) by crossing *DAT-Cre* mice with *Plcg1*^*F/F*^ mice (Fig. 1a). In PLCγ1 cKO mice, the expression of Cre recombinase is driven by a dopamine transporter (DAT) promoter that is specifically activated in dopamine neurons^41–46^. To confirm the knockout of PLCγ1 in dopamine neurons, we immunolabeled midbrain slices with tyrosine hydroxylase (TH) and PLCγ1 antibodies and checked the expression of these proteins in dopamine neurons (Fig. 1b). TH-positive dopamine neurons in control mice (*DAT-IRES-Cre*) normally expressed PLCγ1, but PLCγ1 expression was significantly abolished in the cell bodies of dopamine neurons in both the SNc and VTA regions of PLCγ1 cKO mice, clearly demonstrating that PLCγ1 was selectively eliminated in the dopamine neurons of *DAT-Cre;Plcg1*^*F/F*^ mice (Fig. 1c). Given the very small number of dopamine neurons and relatively large populations of intermingled neighboring nondopamine cells in midbrain tissues, we could not find a selective reduction in PLCγ1 expression in PLCγ1 cKO mice by western blotting (Supplementary Figs. 1a, b and 2b). However, further validation using quantitative real-time PCR revealed the specific downregulation of the PLCγ1 isozyme in PLCγ1 cKO mice (Supplementary Fig. 1c).

Previous studies reported that PLCγ1 is involved in the cellular developmental process^23,47,48^. To check whether the number of dopamine neurons was altered by genetic deletion of PLCγ1, we performed immunohistochemistry and labeled dopamine neurons with TH and DAT antibodies (Fig. 1d). The number of TH-positive dopamine neurons in the SNc and VTA was not changed in PLCγ1 cKO mice compared to control mice (Fig. 1e). In addition, the fluorescence area and expression of TH and DAT, which are responsible for dopamine synthesis and dopamine reuptake, respectively, were normal in both the cell bodies and axons of dopamine neurons in PLCγ1 cKO mice (Fig. 1f–i, Supplementary Fig. 1d–g). We also confirmed that there were no differences in the number of TH-positive dopamine neurons (Supplementary Fig. 1h–m). The general morphology of dopamine axons in the striatum (Supplementary Fig. 1k, n, o) and the detailed morphology of dopamine axons imaged using expansion microscopy were indistinguishable between the genotypes (Supplementary Fig. 1p–t). We further validated this finding by conducting western blot analysis and found that the expression levels of TH and DAT from isolated striatal and midbrain tissues were comparable between the genotypes (Fig. 1j–l, Supplementary Fig. 2a). These results suggest that dopamine neuron-specific PLCγ1 knockout affects neither the population of dopamine neurons nor the expression of key molecular markers of dopamine neurons, including TH and DAT.

### Genetic deletion of PLCγ1 enhances the release of dopamine at dopaminergic terminals

**Fig. 2:**
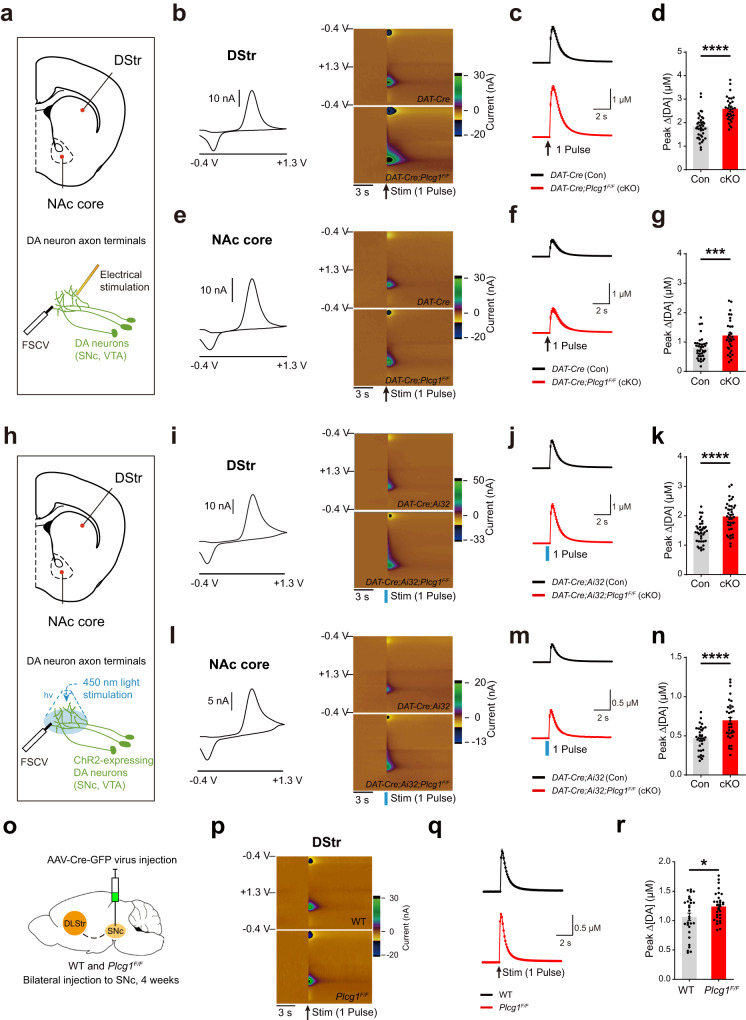
Genetic deletion of PLCγ1 enhances the release of dopamine at dopaminergic terminals. **a** Anatomical location of the DStr and NAc core in a brain slice for fast-scan cyclic voltammetry (FSCV) with electrical stimulation. **b** Representative 2D voltammogram showing oxidation and reduction peaks of dopamine in the DStr (left). Representative 3D color-coded voltammograms from 1 pulse electrical stimulation in the DStr from control and PLCγ1 cKO mice (right). **c** Summary statistics of dopamine release evoked by 1 pulse electrical stimulation in the DStr. **d** Quantification of peak dopamine (DA) amplitude in the DStr by 1 pulse electrical stimulation (unpaired two-tailed *t*-test, Con 1.819 ± 0.085 μM, *n* = 38 slices from 11 mice, cKO 2.573 ± 0.091 μM, *n* = 32 slices from 9 mice, ****p < 0.0001). **e** Representative 2D voltammogram showing oxidation and reduction peaks of dopamine in the NAc core (left). Representative 3D color-coded voltammograms from 1 pulse electrical stimulation in the NAc core from control and PLCγ1 cKO mice (right). **f** Summary statistics of dopamine release evoked by 1 pulse of electrical stimulation in the NAc core. **g** Quantification of peak DA amplitude in the NAc core by 1 pulse electrical stimulation (unpaired two-tailed *t*-test, Con 0.797 ± 0.064 μM, *n* = 35 slices from 11 mice; cKO 1.207 ± 0.102 μM, *n* = 31 slices from 9 mice, ***p = 0.0009). **h** Anatomical location of the DStr and NAc core in a brain slice for FSCV with optogenetic stimulation. **i** Representative 2D voltammogram showing oxidation and reduction peaks of dopamine in the DStr (left). Representative 3D color-coded voltammograms from 1 pulse optogenetic stimulation in the DStr from control and PLCγ1 cKO mice (right). **j** Summary statistics of dopamine release evoked by 1 pulse optogenetic stimulation in the DStr. **k** Quantification of peak DA amplitude in the DStr by 1 pulse optogenetic stimulation (unpaired two-tailed *t*-test, Con 1.442 ± 0.062 μM, *n* = 36 slices from 8 mice, cKO 1.958 ± 0.083 μM, *n* = 39 slices from 8 mice, ****p < 0.0001). **l** Representative 2D voltammogram showing oxidation and reduction peaks of dopamine in the NAc core (left). Representative 3D color-coded voltammograms from 1 pulse optogenetic stimulation in the NAc core from control and PLCγ1 cKO mice (right). **m** Summary statistics of dopamine release evoked by 1 pulse optogenetic stimulation in the NAc core. **n** Quantification of peak DA amplitude in the NAc core by 1 pulse optogenetic stimulation (unpaired two-tailed *t*-test, Con 0.463 ± 0.027 μM, *n* = 34 slices from 8 mice, cKO 0.692 ± 0.043 μM, *n* = 34 slices from 8 mice, ****p < 0.0001). **o** Schematic illustration describing the injection of AAV-Cre-GFP virus bilaterally into each SNc of WT and *Plcg1*^*F/F*^ mice. **p** Representative 3D color-coded voltammograms evoked by 1 pulse electrical stimulation in the DStr from Cre virus-injected brain slices in WT and *Plcg1*^*F/F*^ mice. **q** Summary statistics of dopamine release evoked by 1 pulse electrical stimulation in the DStr. **r** Quantification of peak dopamine (DA) amplitude in the DStr by 1 pulse electrical stimulation (unpaired two-tailed *t*-test, WT 1.063 ± 0.063 μM, *n* = 29 slices from 4 mice, *Plcg1*^*F/F*^1.24 ± 0.042 μM, *n* = 32 slices from four mice, *p = 0.0213).

Dopamine neurons produce dopamine and release it into the synaptic cleft from their axon terminals. To determine whether loss of PLCγ1 influences the physiological functions of dopamine neurons, we first monitored the release of dopamine in the dorsal striatum (DStr) and nucleus accumbens (NAc) core by using fast-scan cyclic voltammetry (FSCV) (Fig. 2a), which can simultaneously measure the amount of dopamine secretion elicited by electrical or optogenetic stimulation and release kinetics^49,50^. Interestingly, dopamine release evoked by either 1 pulse or 5 pulses of electrical stimulation was significantly increased in the dorsal striatum of *DAT-Cre;Plcg1*^*F/F*^ mice (Fig. 2b–d, Supplementary Fig. 3a–c). This enhancement of dopamine release was also detected in the NAc core (Fig. 2e–g, Supplementary Fig. 3d–f), indicating that dopamine release from axon terminals was increased regardless of the specific brain region and that PLCγ1 might be involved in the regulation of dopamine release.

Electrical stimulation in the striatum can stimulate neighboring cholinergic neurons together, which promotes the release of acetylcholine and activates nicotinic acetylcholine receptors on dopaminergic axon terminals, resulting in an increase in dopamine release probability^51,52^. To determine whether this increased dopamine release in the striatum of PLCγ1 cKO mice was affected by electrical stimulation, we produced and checked *DAT-Cre;Ai32;Plcg1*^*F/F*^ mice that express channelrhodopsin-2 (ChR2) with the H134R mutation exclusively in dopamine neurons. As in the case of electrical stimulation, dopamine release elicited by either 1 pulse or 5 pulses of optogenetic stimulation (450 nm wavelength) was markedly elevated both in the dorsal striatum and NAc core of *DAT-Cre;Ai32;Plcg1*^*F/F*^ mice (Fig. 2h–n, Supplementary Fig. 3g–l). These results suggest that dopamine release is facilitated in the striatum by cell type-specific knockout of PLCγ1 in dopamine neurons and that this enhancement of dopamine release from axon terminals is independent of stimulation methods.

Dopamine release might have been affected in PLCγ1 cKO mice by other molecular and cellular changes in dopamine neurons during development. To exclude this possibility and investigate the effect of acute knockout of PLCγ1 at an adult stage in dopamine neurons, AAV-Cre-GFP virus was injected into the SNc region of both WT (C57BL/6J) and *Plcg1*^*F/F*^ adult mice (Fig. 2o). We found that compared to WT mice, dopamine release was also elevated in the dorsal striatum of *Plcg1*^*F/F*^ mice injected with AAV-Cre-GFP virus (Fig. 2p–r).

To further determine whether acute pharmacological inhibition of PLC can elevate dopamine release from dopaminergic axon terminals, we incubated striatal slices with a nonselective PLC inhibitor (U73122, 10 μM) for 30 min and then monitored the release of dopamine. However, acute PLC inhibition by U73122 did not alter dopamine release in the dorsal striatum (Supplementary Fig. 4a–c). Overall, the elevated dopamine release observed in acute PLCγ1 KO and PLCγ1 cKO mice seems to be caused by acute or postdevelopmental chronic deletion of PLCγ1 in dopamine neurons.

GABA and glutamate cotransmission is known to occur at dopamine axon terminals in the striatum. Therefore, we also measured the cotransmission of these fast-acting neurotransmitters to check whether the deletion of PLCγ1 in dopamine neurons affects the release of other neurotransmitters. To our surprise, GABA cotransmission was significantly decreased in PLCγ1 cKO mice (Supplementary Fig. 5a, b), while glutamate cotransmission remained unchanged (Supplementary Fig. 5c, d). It has been reported that aldehyde dehydrogenase 1a1 (ALDH1a1) can synthesize GABA in midbrain dopamine neurons^53^. Therefore, we checked the expression of ALDH1a1 in the dopamine axons of PLCγ1 cKO mice to determine whether the deletion of PLCγ1 leads to the alteration of ALDH1a1. We found that the expression of ALDH1a1 in dopamine axons was significantly decreased in PLCγ1 cKO mice, while the expression of TH in dopamine axons remained unchanged (Supplementary Fig. 5e, f). These results indicate that PLCγ1 might play differential roles in regulating DA transmission and GABA cotransmission at dopamine axon terminals through distinct molecular mechanisms.

### Dendritic morphology and intrinsic properties of dopamine neurons are normal in PLCγ1 cKO mice

**Fig. 3:**
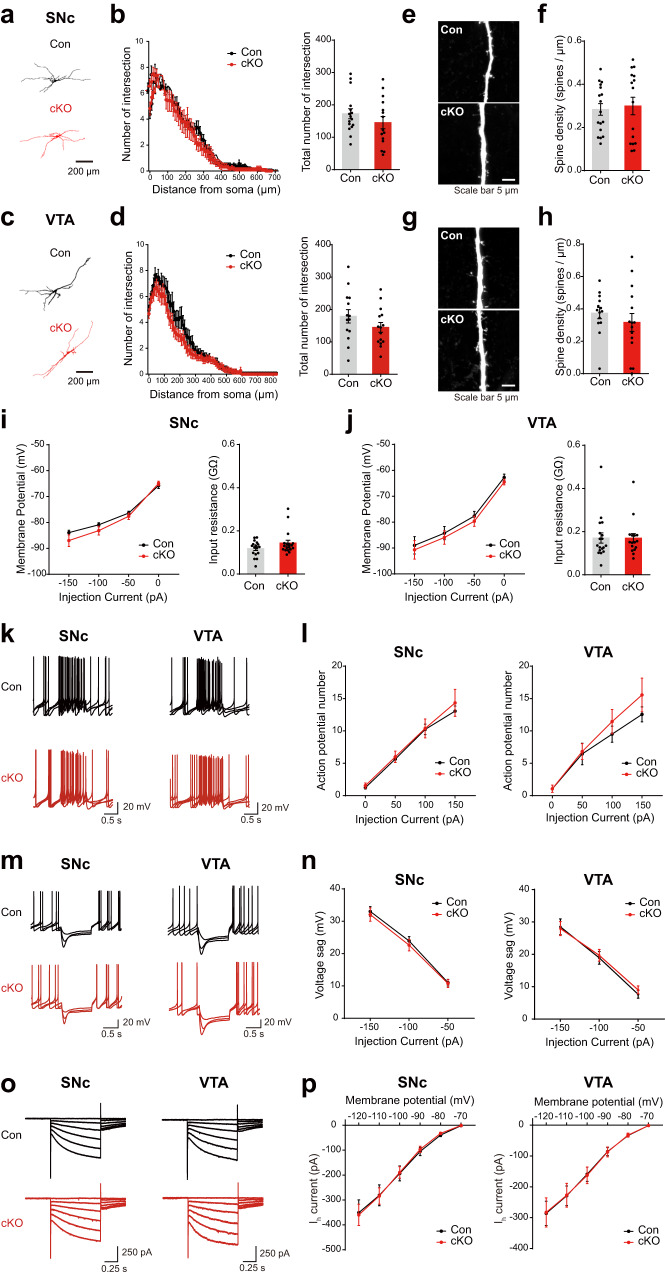
The dendritic morphology and intrinsic properties of dopamine neurons are normal in PLCγ1 cKO mice. **a** Representative dendritic morphology of a single dopamine neuron in the SNc labeled by biocytin. **b** Sholl analysis of the number of dendrite intersections (repeated measures two-way ANOVA, genotype effect, *p* = 0.5497) and the total number of dendritic branches of dopamine neurons in the SNc (unpaired two-tailed *t*-test, Con 172.6 ± 15.53, *n* = 16 cells from 8 mice, cKO 145.1 ± 19.14, *n* = 15 cells from 7 mice, *p* = 0.2721). **c** Representative dendritic morphology of a single dopamine neuron in the VTA labeled by biocytin. **d** Sholl analysis of the number of dendrite intersections (repeated measures two-way ANOVA, genotype effect, *p* = 0.1911) and the total number of dendritic branches of dopamine neurons in the VTA (unpaired two-tailed *t*-test, Con 179.10 ± 20.79, *n* = 14 cells from 8 mice, cKO 145 ± 15.05, *n* = 15 cells from 7 mice, *p* = 0.1911). **e** Representative Z-stack confocal images of dendritic spines on dopamine neurons in the SNc. **f** Dendritic spine density of SNc dopamine neurons (unpaired two-tailed *t*-test, Con 0.283 ± 0.027, *n* = 17 cells from 8 mice, cKO 0.299 ± 0.040, *n* = 16 cells from 7 mice, *p* = 0.7425). **g** Representative Z-stack confocal images of dendritic spines on dopamine neurons in the VTA. **h** Dendritic spine density of VTA dopamine neurons (unpaired two-tailed *t*-test, Con 0.374 ± 0.035, *n* = 14 cells from 8 mice, cKO 0.316 ± 0.055, *n* = 15 cells from 7 mice, *p* = 0.3923). **i** Membrane potential (repeated measures two-way ANOVA, Con *n* = 17 cells from 8 mice, cKO *n* = 18 cells from 7 mice, genotype effect, *p* = 0.3579) and input resistance of SNc dopamine neurons (unpaired two-tailed *t*-test, Con 0.118 ± 0.009 GΩ, *n* = 17 cells from 8 mice, cKO 0.144 ± 0.013 GΩ, *n* = 18 cells from 7 mice, *p* = 0.1184). **j** Membrane potential (repeated measures two-way ANOVA, Con *n* = 18 cells from 8 mice, cKO *n* = 16 cells from 7 mice, genotype effect, *p* = 0.5399) and input resistance of VTA dopamine neurons (unpaired two-tailed *t*-test, Con 0.171 ± 0.024 GΩ, *n* = 18 cells from 8 mice, cKO 0.17 ± 0.021 GΩ, *n* = 16 cells from 7 mice, *p* = 0.9759). **k** Representative recording traces of action potentials from dopamine neurons in the SNc and VTA. **l** Membrane excitability of dopamine neurons in the SNc and VTA (repeated measures two-way ANOVA; SNc Con *n* = 17 cells from 8 mice, cKO *n* = 18 cells from 7 mice, genotype effect, *p* = 0.6927; VTA Con *n* = 18 cells from 8 mice, cKO *n* = 16 cells from 7 mice, genotype effect, *p* = 0.4647). **m** Representative recording traces of voltage sag from SNc and VTA dopamine neurons. **n** Amplitude of voltage sag from SNc and VTA dopamine neurons (repeated measures two-way ANOVA; SNc Con *n* = 17 cells from 8 mice, cKO *n* = 18 cells from 7 mice, genotype effect, *p* = 0.6745; VTA Con *n* = 18 cells from 8 mice, cKO *n* = 16 cells from 7 mice, genotype effect, *p* = 0.8303). **o** Representative recording traces of Ih from SNc and VTA dopamine neurons. **p** Amplitudes of Ih currents from SNc and VTA dopamine neurons (repeated measures two-way ANOVA; SNc Con *n* = 17 cells from 8 mice, cKO *n* = 18 cells from 7 mice, genotype effect, *p* = 0.9644; VTA Con *n* = 18 cells from 8 mice, cKO *n* = 16 cells from 7 mice, genotype effect, *p* = 0.9922).

Synaptic inputs from diverse brain regions are integrated into dopamine neuronal dendrites, the morphological features of which play a significant role in modulating the physiological functions of dopamine neurons^54,55^. To examine whether the genetic deletion of PLCγ1 disturbs the single-cell morphology of dopamine neurons, we injected biocytin into individual dopamine neurons via a recording electrode^56^ and fixed the midbrain slice. After biocytin staining and confocal imaging, we analyzed the dendritic morphology of dopamine neurons by measuring the number of dendritic branches and dendritic spine density. We found that dendritic arborization of dopamine neurons was similar between control and PLCγ1 cKO mice in both the SNc (Fig. 3a, b) and VTA (Fig. 3c, d). Dendritic spine density was also comparable between the genotypes in the SNc (Fig. 3e, f) and VTA (Fig. 3g, h), demonstrating that the dendritic morphology of dopamine neurons is not perturbed by genetic depletion of PLCγ1.

Dopamine neurons generally exhibit unique electrophysiological properties characterized by a long-duration action potential, slow firing activity, pronounced afterhyperpolarization, and hyperpolarization-activated current^57–59^. We next questioned whether any changes in the intrinsic properties of dopamine neurons might contribute to elevated dopamine release in PLCγ1 cKO mice. To measure the intrinsic properties of dopamine neurons, we performed whole-cell patch clamp recording and found that input resistance was normal in PLCγ1 cKO mice (Fig. 3i, j). In addition, disruption of PLCγ1 did not alter the spontaneous firing properties of dopamine neurons, including action potential frequency, peak amplitude, half-width, and threshold (Supplementary Fig. 6a–e). When a depolarizing current was applied to the soma, PLCγ1 cKO mice showed similar neuronal excitability compared with controls (Fig. 3k–l). Hyperpolarization-activated current (Ih) is an inward current generated by hyperpolarization-activated cyclic nucleotide-gated (HCN) cation channels, functionally supporting the pacemaker activity of dopamine neurons^60^. We further investigated the potential differences in voltage sag and Ih current, both of which are mediated by HCN channels in dopamine neurons. However, both voltage sag and Ih were indistinguishable between wild-type control and PLCγ1 cKO mice (Fig. 3m–p). These findings collectively indicate that enhanced dopamine release elicited by genetic deletion of PLCγ1 is not due to any alterations in dendritic morphology and intrinsic electrophysiological properties of dopamine neurons.

### Disruption of PLCγ1 leads to a reduction in inhibitory synaptic transmission in SNc dopamine neurons

**Fig. 4:**
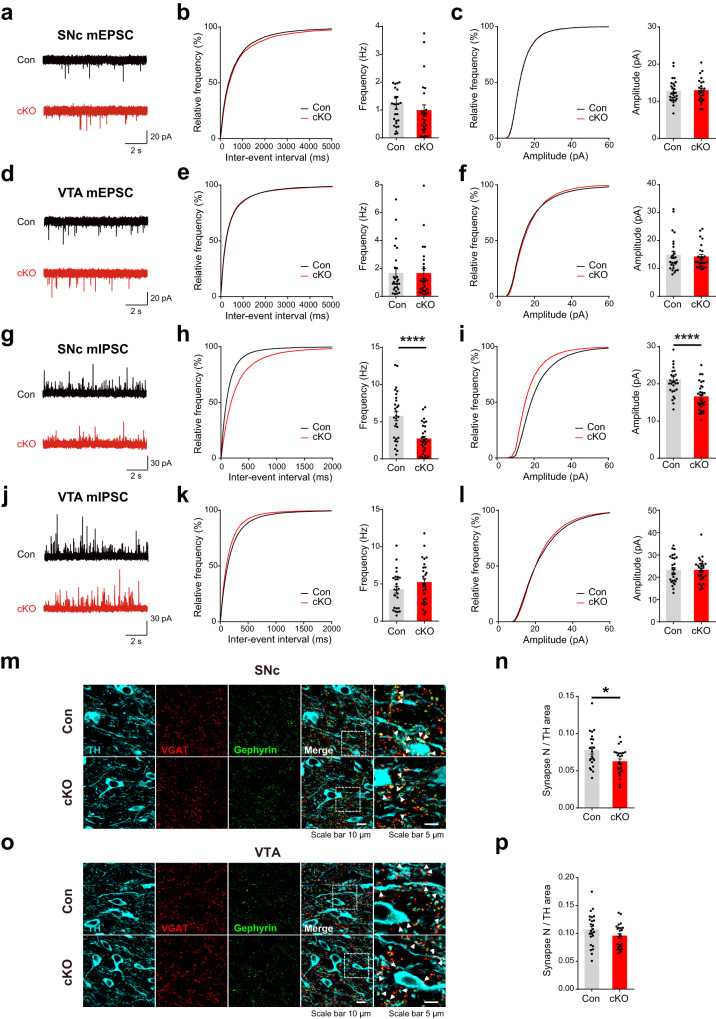
Disruption of PLCγ1 leads to a reduction in inhibitory synaptic transmission in SNc dopamine neurons. **a** Representative mEPSC recording traces from SNc dopamine neurons. **b**, **c** Cumulative graphs and summary statistics for the frequency (**b**) and amplitude (**c**) of mEPSCs from SNc dopamine neurons (unpaired two-tailed *t*-test, Con *n* = 27 cells from six mice, cKO *n* = 25 cells from six mice; Con 1.257 ± 0.235 Hz, cKO 0.984 ± 0.201 Hz, *p* = 0.3837; Con 12.69 ± 0.593 pA, cKO 12.92 ± 0.629 pA, *p* = 0.7893). **d** Representative mEPSC recording traces from VTA dopamine neurons. **e**, **f** Cumulative graphs and summary statistics for the frequency (**e**) and amplitude (**f**) of mEPSCs from VTA dopamine neurons (unpaired two-tailed *t*-test, Con *n* = 27 cells from six mice, cKO *n* = 26 cells from seven mice; Con 1.657 ± 0.329 Hz, cKO 1.641 ± 0.355 Hz, *p* = 0.9748; Con 14.81 ± 1.191 pA, cKO 14.15 ± 0.828 pA, *p* = 0.6507). **g** Representative mIPSC recording traces from SNc dopamine neurons. **h**, **i** Cumulative graphs and summary statistics for the frequency (**h**) and amplitude (**i**) of mIPSCs from SNc dopamine neurons (unpaired two-tailed *t*-test, Con *n* = 29 cells from 8 mice, cKO *n* = 30 cells from eight mice; Con 5.736 ± 0.604 Hz, cKO 2.686 ± 0.359 Hz, ****p < 0.0001; Con 21.14 ± 0.818 pA, cKO 16.54 ± 0.675 pA, ****p < 0.0001). **j** Representative mIPSC recording traces from VTA dopamine neurons. **k**, **l** Cumulative graphs and summary statistics for the frequency (**k**) and amplitude (**l**) of mIPSCs from VTA dopamine neurons (unpaired two-tailed *t*-test, Con *n* = 27 cells from 8 mice, cKO *n* = 29 cells from 9 mice; Con 4.239 ± 0.461 Hz, cKO 5.208 ± 0.516 Hz, *p* = 0.1689; Con 23.18 ± 1.188 pA, cKO 23.11 ± 0.955 pA, *p* = 0.9658). **m**, **o** Representative confocal images of inhibitory synapses in the SNc (**m**) and VTA (**o**). **n**, **p** Colocalization of VGAT and TH-positive gephyrin per TH area in the SNc (**n**) and VTA (**p**) (unpaired two-tailed *t*-test, *n* = 24 images from four mice per genotype; SNc, Con 0.077 ± 0.005, cKO 0.062 ± 0.004, *p = 0.0158; VTA, Con 0.107 ± 0.006, cKO 0.095 ± 0.004, *p* = 0.1255).

Altered synaptic transmission to dopamine neurons can influence the properties of neuronal firing and subsequent dopamine release^38,61^. To assess any potential changes in the synaptic transmission to dopamine neurons, we checked spontaneous miniature excitatory and inhibitory synaptic transmission in the SNc and VTA. However, the frequency and amplitude of miniature excitatory postsynaptic currents (mEPSCs) monitored in the SNc dopamine neurons of PLCγ1 cKO mice were not different from those in control mice (Fig. 4a–c). Furthermore, similar findings were made in the dopamine neurons of the VTA, confirming that excitatory synaptic transmission to dopamine neurons is not altered by genetic deletion of PLCγ1 (Fig. 4d–f). We then turned our attention to inhibitory synaptic transmission to dopamine neurons and recorded miniature inhibitory postsynaptic currents (mIPSCs). Notably, the amplitude and frequency of mIPSCs were significantly attenuated in the SNc dopamine neurons of PLCγ1 cKO mice (Fig. 4g–i), while there was no difference between the genotypes in the physiological features of mIPSCs in VTA dopamine neurons (Fig. 4j–l).

The deletion of PLCγ1 in postsynaptic dopamine neurons may affect inhibitory synapse number, which can lead to the attenuation of mIPSC frequency. To check this possibility, we analyzed the number of GABAergic synapses contacting SNc and VTA dopamine neurons. We used vesicular GABA transporter (VGAT) as an inhibitory presynaptic marker and the colocalization of TH and gephyrin as an inhibitory postsynaptic marker (Fig. 4m, o). Interestingly, the number of inhibitory synapses entering dopamine neurons (triple colocalization of VGAT, TH, and gephyrin) was markedly reduced in the SNc of PLCγ1 cKO mice (Fig. 4m, n), whereas no alteration was observed in the VTA (Fig. 4o, p). Our findings indicate that selective reduction of inhibitory synaptic transmission to dopamine neurons, specifically in the SNc, might partially contribute to facilitated dopamine release in PLCγ1 cKO mice.

### Enhanced dopamine release in PLCγ1 cKO mice does not exhibit a protective effect on the neurodegeneration induced by 6-OHDA

Given the vulnerability of SNc dopamine neurons in Parkinson’s disease (PD) and their premature degeneration during disease progression^62,63^, we hypothesized that the increased dopamine release in PLCγ1 cKO mice would attenuate the degeneration of dopamine neurons in an animal model of PD. To validate this possibility, we injected 6-OHDA (6-hydroxydopamine) unilaterally into the medial forebrain bundle (MFB) of *DAT-Cre* and *DAT-Cre;Plcg1*^*F/F*^ mice (Supplementary Fig. 7a). Three days after injection, 6-OHDA induced significant degeneration of TH- and DAT-positive axonal processes in the ipsilateral DStr of wild-type control and PLCγ1 cKO mice (Supplementary Fig. 7b). However, the extent of axonal degeneration found in PLCγ1 cKO mice was comparable to that in wild-type control mice (Supplementary Fig. 7c, d).

To further examine the potential protective effect of PLCγ1 knockout on PD-like pathology, we measured the release of dopamine in the ipsilateral DStr of wild-type control and PLCγ1 cKO mice injected with 6-OHDA. Consistent with our previous results, dopamine release by electrical stimulation was increased in the contralateral DStr of PLCγ1 cKO mice compared to the control group (Supplementary Fig. 7e–j). Nevertheless, these heightened levels of dopamine release in PLCγ1 cKO mice were unable to exert a protective effect against functional deterioration following 6-OHDA injection, as dopamine release was significantly diminished in the ipsilateral DStr of both *DAT-Cre* and *DAT-Cre;Plcg1*^*F/F*^ mice (Supplementary Fig. 7e–j). Collectively, these results indicate that enhanced dopamine release by the deletion of PLCγ1 in dopamine neurons does not mitigate the neurodegeneration induced in an animal model of PD.

### Enhanced localization of VMAT2 in dopaminergic axons may facilitate DA release in PLCγ1 cKO mice

**Fig. 5:**
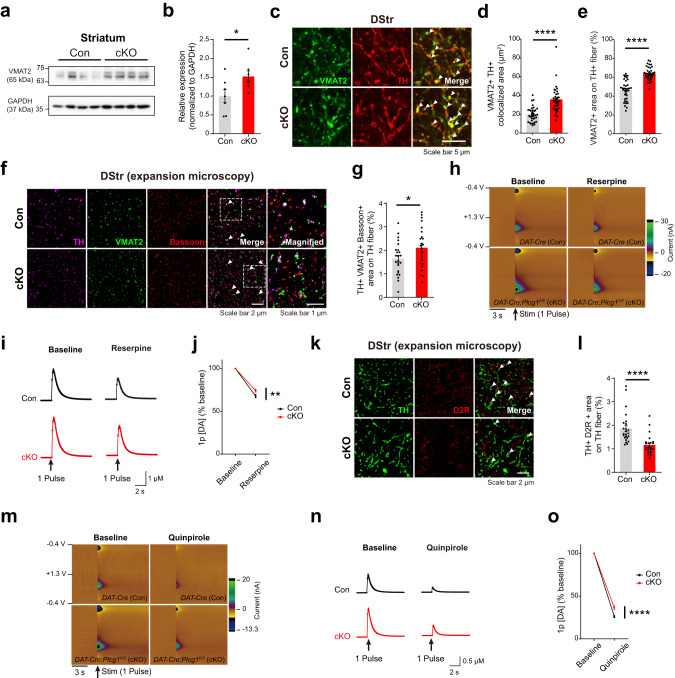
Enhanced localization of VMAT2 in dopaminergic axons may facilitate DA release in PLCγ1 cKO mice. **a** Representative western blot analysis of VMAT2 expression in striatal synaptosomes from control and PLCγ1 cKO mice. **b** Quantification of VMAT2 expression in striatal synaptosomes from western blot analysis (unpaired two-tailed *t*-test, *n* = 7 mice per genotype; Con 1 ± 0.175, cKO 1.521 ± 0.146, *p = 0.0413). **c** Representative confocal images of VMAT2 expression in striatal dopaminergic axons (white arrowhead: colocalized area). **d** VMAT2 and TH colocalized area (unpaired two-tailed *t*-test, *n* = 36 images from four mice per genotype; Con 19.99 ± 1.283 μm^2^, cKO 35.67 ± 2.3 μm^2^, ****p < 0.0001). **e** Percentage of VMAT-positive area on TH-positive axons (unpaired two-tailed *t*-test, *n* = 36 images from four mice per genotype; Con 46.31 ± 1.798%, cKO 63.98 ± 1.154%, ****p < 0.0001). **f** Representative expansion microscopy confocal images of dopamine axon terminals in the DStr labeled by TH, VMAT2, and bassoon (white arrowhead: triple-colocalized area, expansion factor = 5). **g** Summary statistics for the triple colocalization area of TH, VMAT2, and bassoon on TH fibers (unpaired two-tailed *t*-test, *n* = 24 images from four mice per group; Con 1.643 ± 0.141%, cKO 2.108 ± 0.175%, *p = 0.0447). **h** Representative 3D color-coded voltammograms by 1 pulse electrical stimulation in the DStr of control and PLCγ1 cKO mice before and after treatment with reserpine (3 μM). **i** Summary statistics of dopamine release evoked by 1 pulse electrical stimulation in the DStr before and after treatment with reserpine. **j** Reduction in peak DA release before and after treatment with reserpine (repeated measures two-way ANOVA, Con *n* = 18 slices from 3 mice, cKO *n* = 16 slices from three mice, genotype effect, *p = 0.0224, Sidak’s multiple comparisons test, **p = 0.0024). **k** Representative expansion microscopy confocal images of D2R on dopamine axons in the DStr labeled by TH and D2R (white arrowhead: colocalized area, expansion factor = 5). **l** Summary statistics for the colocalization area of TH and D2R on TH fibers (unpaired two-tailed *t*-test, *n* = 24 images from four mice per genotype; Con 1.864 ± 0.134, cKO 1.162 ± 0.083, ****p < 0.0001). **m** Representative 3D color-coded voltammograms by 1 pulse electrical stimulation in the DStr of control and PLCγ1 cKO mice before and after treatment with quinpirole (50 nM). **n** Summary statistics of dopamine release evoked by 1 pulse electrical stimulation in the DStr before and after treatment with quinpirole. **o** Reduction in peak DA release before and after treatment with quinpirole (repeated measures two-way ANOVA, Con *n* = 15 slices from 3 mice, cKO *n* = 16 slices from three mice, genotype effect, ***p = 0.0007, Sidak’s multiple comparisons test, ****p < 0.0001).

Vesicular monoamine transporter 2 (VMAT2) is a key presynaptic regulator in dopamine release, packaging cytosolic dopamine into synaptic vesicles^64,65^. Increased expression and function of VMAT2 can directly lead to elevated dopamine release in the striatum^66^, yet it is unclear whether the loss of PLCγ1 affects the expression of VMAT2 in dopaminergic axons. To answer this question, we separated striatal synaptosomal fractions and performed western blotting to examine the level of VMAT2 (Fig. 5a, b, Supplementary Fig. 2c). Indeed, we found a significant increase in VMAT2 expression in PLCγ1 cKO mice (Fig. 5b). We also co-labeled striatal tissues with VMAT2 and TH antibodies and checked the colocalization of these proteins in the dorsal striatum (Fig. 5c). We found that the localization of VMAT2 on TH-positive dopaminergic axons was markedly increased by conditional KO of PLCγ1 in dopamine neurons (Fig. 5d, e).

Utilizing expansion microscopy, we further imaged and analyzed VMAT2 localization with a higher resolution to check whether VMAT2 is specifically colocalized in dopamine varicosities^37,67,68^ (Supplementary Fig. 1p). In this experiment, we also examined the presynaptic scaffold bassoon, which can contribute to the establishment of active zone-like structures in dopamine axons^69^. Our expansion microscopy results revealed that the triple colocalization of these molecules in the dorsal striatum was markedly increased in PLCγ1 cKO mice (Fig. 5f, g). These results demonstrate that VMAT2 is not universally upregulated throughout dopaminergic axons but rather specifically increased in dopaminergic varicosities.

To investigate whether increased colocalization of VMAT2 in the dopaminergic axons of PLCγ1 cKO mice functionally influences dopamine release, we compared the effect of reserpine, a competitive VMAT2 inhibitor that can block the uptake of dopamine to synaptic vesicles, on dopamine release between the genotypes. In the FSCV experiments, a subsaturating concentration of reserpine (3 µM) considerably attenuated dopamine release in both control and PLCγ1 cKO mice (Fig. 5h–j). However, reserpine treatment in striatal slices from PLCγ1 cKO mice suppressed dopamine release to a lesser extent than that in control mice, thus strengthening the idea that enhanced localization of VMAT2 in dopaminergic axons could functionally support the facilitation of dopamine release in PLCγ1 cKO mice (Fig. 5h–j).

Dopamine release is also modulated by extracellular GABA and presynaptic GABA receptors^70,71^. GABA_A_ α1 subunit-containing receptors are highly expressed in the striatum, and dopamine neurons mainly express the α1 subunit of GABA_A_ receptors^72,73^. Moreover, PLCγ1 seems to regulate the surface expression of the GABA_A_ α1 subunit^74,75^. Therefore, we evaluated the localization of the GABA_A_ α1 subunit on dopaminergic axons in the dorsal striatum but found that disruption of PLCγ1 does not affect the localization of the GABA_A_ α1 subunit on dopaminergic axons, indicating that a GABA_A_ receptor-mediated mechanism may not be responsible for enhanced dopamine release in PLCγ1 cKO mice (Supplementary Fig. 8a–c).

Additionally, dopamine D2 autoreceptor (D2R) is commonly expressed in dopaminergic axon terminals and provides negative feedback to regulate excessive extracellular dopamine levels^76^. To better visualize the presynaptic D2R expressed in dopamine axons, we utilized expansion microscopy again (Fig. 5k). We found that the colocalization of D2R on TH-positive dopaminergic axons was significantly decreased in PLCγ1 cKO mice, potentially suggesting the attenuation of presynaptic inhibition through D2R (Fig. 5l). To prove this possibility, we further compared the effect of the selective D2R agonist quinpirole (50 nM) on dopamine release between control and PLCγ1 cKO mice. Notably, the suppression of dopamine release by quinpirole was less effective in PLCγ1 cKO mice than in control mice (Fig. 5m–o). These results demonstrate that the loss of PLCγ1 in dopamine neurons decreases the colocalization of D2R on dopaminergic axons, and this reduction in D2R colocalization leads to the attenuation of D2R-mediated presynaptic inhibition of dopamine release.

### Altered synapsin III expression might underlie the increased localization of VMAT2 in the dopaminergic axons of PLCγ1 cKO mice

**Fig. 6:**
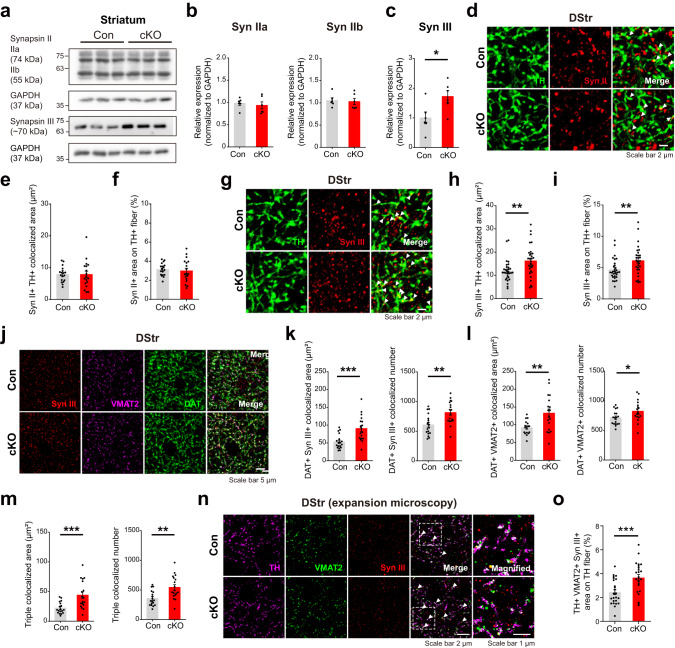
Altered synapsin III expression might underlie the increased localization of VMAT2 in the dopaminergic axons of PLCγ1 cKO mice. **a** Representative western blot images of synapsin II and III expression in striatal synaptosomes from control and PLCγ1 cKO mice. **b**, **c** Quantification of synapsin IIa and IIb (**b**) and synapsin III (**c**) expression in the striatum (unpaired two-tailed *t*-test, *n* = 6 mice per genotype; Syn IIa, Con 1 ± 0.05, cKO 0.9481 ± 0.072, *p* = 0.5674; Syn IIb, Con 1 ± 0.048, cKO 0.9824 ± 0.084, *p* = 0.8599; Syn III, Con 1 ± 0.199, cKO 1.722 ± 0.200, *p = 0.0282). **d** Representative confocal images showing the expression of synapsin II in striatal dopaminergic axons (white arrowhead: colocalized area). **e** Synapsin II and TH colocalized area in the DStr (unpaired two-tailed *t*-test, *n* = 18 images from three mice per genotype; Con 7.873 ± 0.560 μm^2^, cKO 7.869 ± 1.020 μm^2^, *p* = 0.9974). **f** Percentage of synapsin II-positive area on TH-positive axons in the DStr (unpaired two-tailed *t*-test, *n* = 18 images from three mice per genotype; Con 3.132 ± 0.156%, cKO 2.985 ± 0.283%, *p* = 0.6534). **g** Representative confocal images showing the expression of synapsin III in striatal dopaminergic axons (white arrowhead: colocalized area). **h** Synapsin III and TH colocalized area in the DStr (unpaired two-tailed *t*-test, *n* = 30 images from five mice per genotype; Con 11.88 ± 0.831 μm^2^, cKO 16.23 ± 0.123 μm^2^, **p = 0.0049). **i** Percentage of synapsin III-positive area on TH-positive axons in the DStr (unpaired two-tailed *t*-test, *n* = 30 images from five mice per genotype; Con 4.688 ± 0.302%, cKO 6.113 ± 0.416%, **p = 0.0075). **j** Representative confocal images of triple colocalization (synapsin III, VMAT2, and DAT) in the striatum. **k** Colocalization of DAT and synapsin III in the DStr (unpaired two-tailed *t*-test, *n* = 18 images from three mice per genotype; area, Con 53.06 ± 4.732 μm^2^, cKO 90.41 ± 8.287 μm^2^, ***p = 0.0004; number, Con 607.7 ± 40.71 puncta, cKO 814.8 ± 45.35 puncta, **p = 0.0017). **l** Colocalization of DAT and VMAT2 in the DStr (unpaired two-tailed *t* test, *n* = 18 images from three mice per genotype; area, Con 92.23 ± 4.504 μm^2^, cKO 132.8 ± 11.81 μm^2^, **p = 0.0029; number, Con 711.9 ± 28.95 puncta, cKO 823.7 ± 40.93 puncta, *p = 0.0326). **m** Triple colocalization of synapsin III, VMAT2, and DAT in the DStr (unpaired two-tailed *t*-test, *n* = 18 images from three mice per genotype; area, Con 22.3 ± 2.274 μm^2^, cKO 44.02 ± 5.456 μm^2^, ***p = 0.0008; number, Con 358.4 ± 29.72 puncta, cKO 544 ± 45.89 puncta, **p = 0.0018). **n** Representative expansion microscopy confocal images of dopamine axon terminals in the DStr labeled by TH, VMAT2, and synapsin III (white arrowhead: triple-colocalized area, expansion factor = 5). **o** Summary statistics for the triple colocalization area of TH, VMAT2, and synapsin III on TH fibers (unpaired two-tailed *t*-test, *n* = 24 images from four mice per group; Con 2.445 ± 0.214%, cKO 3.663 ± 0.264%, ***p = 0.0008).

Synapsin is a presynaptic protein abundant in most synapses in the brain that is critical for the regulation of the number of synaptic vesicles in the active zone by controlling the localization of synaptic vesicles between the reserve pool and the readily releasable pool^77–79^. Synapsins are encoded by three genes in mice and humans: synapsin I, II, and III. In addition, alternative splicing of these genes can generate several synapsin isoforms. Synapsin isoforms are differentially expressed in the nerve terminals of various types of neurons, acting as key regulators of presynaptic vesicle release. Synapsin I, II, and III begin to be highly expressed during the developmental stages in the brain^78,80^. Interestingly, synapsin III is reported to modulate the release of dopamine^81,82^. Furthermore, PLCγ1 seems to interact with synapsins^83,84^. Given these previous findings, it is natural to speculate that any alterations in synapsin may contribute to the increased localization of VMAT2 on dopaminergic axons in PLCγ1 cKO mice. Thus, we first assessed the expression of synapsin II and synapsin III in the striatum by conducting western blot analysis (Fig. 6a, Supplementary Fig. 2d). To minimize the effect of synapsins expressed in nonsynaptic compartments in the striatum, we examined the expression of synapsins in synaptosomal fractions from the striatal tissue. We found that the expression of synapsin III was remarkably elevated in striatal synaptosomes of PLCγ1 cKO mice, while synapsin II levels were not affected by deletion of PLCγ1 (Fig. 6b, c).

To more specifically interrogate the altered expression of synapsin II and synapsin III on dopaminergic axons, we performed immunohistochemistry targeting synapsin II and synapsin III in the dorsal striatum. As in the case of western blot analysis, the expression of synapsin II, which is responsible for glutamatergic vesicle release, was not changed by disruption of PLCγ1 (Fig. 6d–f). However, the expression of synapsin III was significantly increased in the dopaminergic axons of PLCγ1 cKO mice (Fig. 6g–i, Supplementary Fig. 9a). Synapsin III has domains similar to those of synapsin I and II, and the alteration of synapsin III can affect other synapsin proteins. To identify any compensatory changes in synapsin I in PLCγ1 cKO mice, we examined synapsin I expression in synaptosomal fractions in the striatum. We found that the expression of synapsin I was not altered in the striatum of PLCγ1 cKO mice (Supplementary Fig. 10). 3D stack images of synapsins also showed enhanced colocalization of synapsin III on dopaminergic axons (Supplementary Fig. 9b–e). To further investigate whether PLCγ1 can regulate the localization of synapsin III and VMAT2 at dopamine terminals for dopamine release, we checked the triple colocalization of DAT, synapsin III, and VMAT2 in the striatum. Consistent with our previous results, synapsin III and VMAT2 were significantly increased in DAT-positive dopaminergic axons by deletion of PLCγ1 (Fig. 6j–l). Furthermore, the triple colocalization of DAT, synapsin III, and VMAT2 was elevated in the striatum by PLCγ1 cKO (Fig. 6m). To confirm whether the increased colocalization of synapsin III in the striatum is also more limited to dopamine terminals, we performed expansion microscopy, which can clearly capture the triple colocalization of TH, synapsin III, and VMAT2 (Fig. 6n, Supplementary Fig. 1p). We found that the triple colocalization of TH, synapsin III, and VMAT2 was upregulated in PLCγ1 cKO mice, validating that synapsin III is specifically upregulated in dopamine terminals (Fig. 6o). Taken together, our results suggest that deficiency of PLCγ1 in dopamine neurons may facilitate dopamine release by promoting synapsin III expression and concomitant localization of VMAT2-positive synaptic vesicles in dopaminergic axon terminals.

### Attenuated dopamine release by the knockdown of VMAT2 and synapsin III is alleviated in PLCγ1 cKO mice

**Fig. 7:**
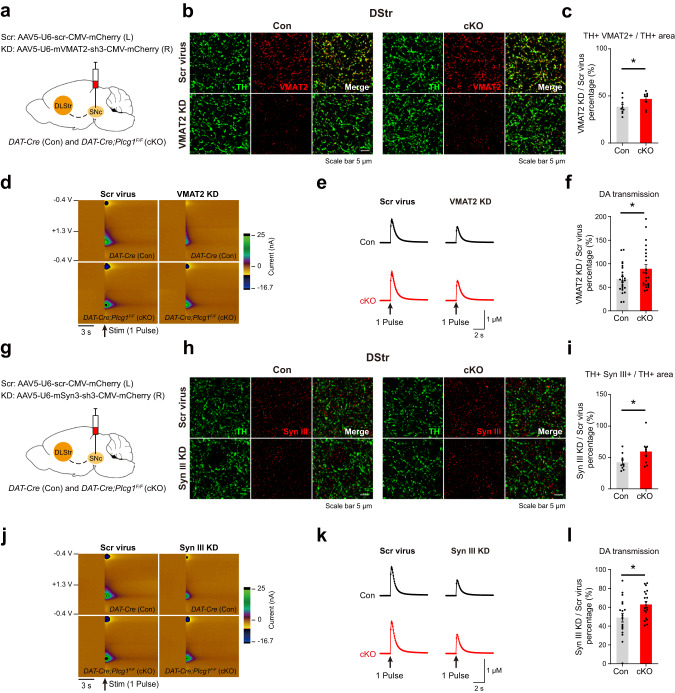
Attenuated dopamine release by the knockdown of VMAT2 and synapsin III is alleviated in PLCγ1 cKO mice. **a** Schematic illustration describing the injection of Scr (scrambled) virus and VMAT2 KD virus into each SNc (left hemisphere: Scr virus, right hemisphere: KD virus) of *DAT-Cre* and *DAT-Cre;Plcg1*^*F/F*^ mice. **b** Representative confocal images of TH and VMAT2 in the DStr of each hemisphere of *DAT-Cre* and *DAT-Cre;Plcg1*^*F/F*^ mice. **c** Summary statistics for the VMAT2 area on TH fibers in the VMAT2 KD group compared to the Scr virus group (unpaired two-tailed *t*-test, *n* = 9 images from three mice per group; Con 38.51 ± 2.708%, cKO 46.75 ± 2.661%, *p = 0.0453). **d** Representative 3D color-coded voltammograms from 1 pulse electrical stimulation in the DStr of Scr virus and VMAT2 KD brain slices in control and PLCγ1 cKO mice. **e** Summary statistics of dopamine release evoked by 1 pulse electrical stimulation in the DStr. **f** Quantification of the peak dopamine amplitude of VMAT2 KD mice compared to Scr virus mice in the DStr (unpaired two-tailed t-test, *n* = 24 slices from five mice per group; Con 67.26 ± 6.063%, cKO 89.36 ± 8.877%, *p = 0.0455). **g** Schematic illustration describing the injection of Scr virus and synapsin III KD virus into each SNc (left hemisphere: Scr virus, right hemisphere: KD virus) of *DAT-Cre* and *DAT-Cre;Plcg1*^*F/F*^ mice. **h** Representative confocal images of TH and synapsin III in the DStr of each hemisphere of *DAT-Cre* and *DAT-Cre;Plcg1*^*F/F*^ mice. **i** Summary statistics for the synapsin III area on TH fibers in synapsin III KD compared to Scr virus (unpaired two-tailed *t*-test, *n* = 10 images from four mice per group; Con 42.37 ± 3.937%, cKO 59.42 ± 6.441%, *p = 0.0365). **j** Representative 3D color-coded voltammograms from 1 pulse electrical stimulation in the DStr of Scr virus and synapsin III KD brain slices in control and PLCγ1 cKO mice. **k** Summary statistics of dopamine release evoked by 1 pulse of electrical stimulation in the DStr. **l** Quantification of peak dopamine amplitude of synapsin III KD compared to Scr virus in the DStr by 1 pulse electrical stimulation (unpaired two-tailed *t*-test, *n* = 20 slices from four mice per group; Con 48.99 ± 4.682%, cKO 62.96 ± 3.230%, *p = 0.0179).

To provide more evidence supporting the potential role of VMAT2 and synapsin III in facilitating dopamine release in PLCγ1 cKO mice, we knocked down the expression of VMAT2 and synapsin III in dopamine neurons by injecting AAV expressing shRNA against VMAT2 and synapsin III. We first examined the expression level of VMAT2 in dopamine axons after knockdown and confirmed that VMAT2 was successfully knocked down in the dopamine neurons of wild-type control and PLCγ1 cKO mice (Fig. 7a–c). Importantly, we found that the remaining VMAT2 in dopamine neurons was significantly higher in PLCγ1 cKO mice (Fig. 7b, c). Consistent with the results of reserpine treatment, VMAT2 knockdown also considerably decreased dopamine release in both wild-type control and PLCγ1 cKO mice (Fig. 7d, e), while this attenuation in dopamine release was less pronounced in PLCγ1 cKO mice, mimicking the effect of reserpine (Fig. 7d–f). Then, we knocked down synapsin III and found that the expression of synapsin III was greatly reduced in the dopamine axons of wild-type control and PLCγ1 cKO mice, while the surviving synapsin III level was higher in PLCγ1 cKO mice (Fig. 7g–i). As in the case of VMAT2 knockdown, the knockdown of synapsin III in dopamine neurons considerably weakened dopamine transmission in the dorsal striatum of wild-type control and PLCγ1 cKO mice (Fig. 7j, k). Furthermore, dopamine release in PLCγ1 cKO mice was more mildly affected by synapsin III knockdown than in wild-type control mice (Fig. 7j–l). Taken together, our results indicate that the elevated levels of both VMAT2 and synapsin III in DA terminals might contribute to the facilitation of DA transmission in PLCγ1 cKO mice.

Finally, to further search for the potential molecular mechanisms behind this enhanced dopamine release in PLCγ1 cKO mice, we investigated whether the level of PIP_2_ was altered in PLCγ1 cKO mice and measured the level of PIP_2_ in dopamine neurons. PIP_2_ plays an important role in vesicular release and synaptic vesicle recycling^85,86^. Notably, we observed a significant increase in PIP_2_ levels in the dopamine neurons of PLCγ1 cKO mice (Supplementary Fig. 11a–c). This result indicates that elevated PIP_2_ levels caused by PLCγ1 deletion in dopamine neurons could be implicated in the regulation of dopamine release via VMAT2/synapsin III, eventually leading to enhanced dopamine release.

## Discussion

In this study, we sought to investigate the physiological functions of PLCγ1 in dopamine neurons by generating and examining cell type-specific PLCγ1 conditional knockout mice. We found that genetic deletion of PLCγ1 significantly increases dopamine release without affecting the cell number and cellular morphology of dopamine neurons. Elevated dopamine release does not seem to be caused by any alterations in dopamine synthesis, reuptake, or intrinsic membrane properties, but inhibitory synaptic transmission to dopamine neurons was markedly reduced in PLCγ1 cKO mice, potentially lowering the threshold for synaptically driven action potentials in dopamine neurons. Most importantly, localization of both VMAT2 and synapsin III in dopaminergic axons was noticeably increased by disruption of PLCγ1. This enhanced localization, especially in dopamine terminals, was further validated by expansion microscopy with better spatial resolution. The knockdown of VMAT2 and synapsin III in dopamine neurons caused a sharp decrease in dopamine release, while this attenuation in dopamine release was less pronounced in PLCγ1 cKO mice. Considering the key role of synapsin in synaptic vesicle trafficking, the deletion of PLCγ1 might facilitate the trafficking of VMAT2-positive synaptic vesicles to dopaminergic axon terminals via a synapsin III-mediated mechanism.

Previous studies have suggested that PLC is implicated in the exocytosis of neurotransmitters. Glutamate release induced by BDNF is mediated by PLC/IP_3_-dependent Ca^2+^ signaling in cerebellar neurons^87^. Moreover, this well-known PLC signaling is required to promote the enhancement of GABA release, which is induced by ethanol in Purkinje cells in the cerebellum^88^. On the other hand, inhibition of PLC by U73122 blocks PIP_2_ hydrolysis and Ca^2+^ release, resulting in the suppression of exocytosis of vesicles in mast cells, neuroendocrine cells, and hippocampal neurons^89,90^. Despite these findings, it is apparent that in our study, treatment with a PLC inhibitor did not change dopamine release from dopamine neurons, suggesting that the elevated dopamine release found in PLCγ1 cKO mice may not be due to Ca^2+^-dependent short-term modulation of exocytosis. However, considering the nonspecific nature of U73122 in the inhibition of PLC isozymes, a more specific study is necessary to fully resolve this issue. PLC signaling can regulate synaptic vesicles via interaction with synaptic release machinery. The generation of DAG by activating PLC can stimulate vesicle release via Munc13-1, which is essential for synaptic vesicle fusion in chromaffin cells^91,92^. DAG is also able to activate protein kinase C (PKC), which controls the docking, priming, and fusion process of synaptic vesicles^93,94^. In addition to these functions, PLC can limit synaptic dopamine availability by controlling the endocytic process of receptors and transporters. Activation of PKC, which is a major downstream molecule of PLC, can promote DAT and D2R internalization in cultured dopamine neurons^95,96^. Moreover, overexpression of PLCγ1 increases clathrin-mediated endocytosis of EGF receptors in PC12 cells^97,98^. However, in our data, the expression of DATs in dopaminergic axons was not altered by deletion of PLCγ1, whereas D2R expression on TH-positive dopaminergic axons was downregulated. Overall, it is possible that the role of PLC in the regulation of neurotransmitter release greatly varies depending on both the particular cell types where PLC is expressed and the specific isoform of PLC.

It is important to note that brain-derived neurotrophic factor (BDNF) and its receptor TrkB (tropomyosin receptor kinase B), both of which are upstream activators of PLCγ1, are related to dopamine release. According to several studies using in vivo microdialysis and slice voltammetry, extracellular dopamine levels and dopamine release were increased together in BDNF heterozygous mice^99–101^, while one other study reported a contrasting result^102^. In our study, the elevated dopamine release observed in PLCγ1 cKO mice does not appear to be caused by alterations in dopamine synthesis and reuptake. Instead, elevated localization of VMAT2 and synapsin III in dopaminergic axons might underlie the enhancement of dopamine release caused by the deletion of PLCγ1. Synapsin is one of the critical regulators that govern the mobilization of synaptic vesicles to release sites^77,103^. Synapsins are encoded by three genes (I, II, and III) in mammals, and among them, synapsin III has been shown to exert a regulatory effect on dopamine neurons^81,82^. Double knockout of synapsin I and synapsin II led to decreased glutamate and GABA transport into synaptic vesicles, but vesicular uptake of dopamine was unaffected^104^. Noticeably, synapsin III single knockout mice showed increased dopamine release^81^, which is in contrast to our findings, and the reasons for this discrepancy are not clear. When we acutely knocked down synapsin III in dopamine neurons, we found that the remaining colocalization of TH and synapsin III over the TH-positive axonal area was significantly higher in PLCγ1 cKO mice than in control mice. Importantly, contrary to the previous finding, we found that the knockdown of synapsin III in dopamine neurons markedly weakens DA transmission in the DStr. Furthermore, the remaining DA transmission in PLCγ1 cKO mice was significantly higher than that in wild-type control mice. Our findings suggest the following possibilities. First, in addition to the previous finding about the role of synapsin III in dopamine transmission^81^, the functional roles of synapsin III in regulating DA transmission can be multifaceted; the specific roles of synapsin III in DA terminals and transmission remain poorly understood. Synapsin III has also been shown to have versatile functions across various neuronal cell types and differentially regulates neurotransmitter release in excitatory and inhibitory neurons^105,106^. According to one study, synapsin III can enhance neurotransmitter release probability, which partially supports our findings^106^. In our experiment, we acutely knocked down the expression of synapsin III, which subsequently led to a reduction in dopamine transmission. Thus, it is likely that the regulatory role of synapsin III can vary depending on the physiological circumstances at dopamine terminals. Moreover, there are notable differences in the experimental conditions between our findings and the previous one. The previous study used whole-body knockout mice in which synapsin III was widely knocked out during brain development, while we acutely knocked down synapsin III in the dopamine neurons of adult mice^81^. In addition, the genetic background of control and PLCγ1 cKO mice in our study was C57BL/6J, but the previous study did not provide specific information on the genetic background of synapsin III knockout mice; it is well known that the genetic background of mice can critically affect the physiological functions of many synaptic proteins expressed in both presynaptic and postsynaptic sites. In summary, all of these factors could contribute to the discrepancy between our findings and the previous report. It is worth mentioning that similar to the results from the VMAT2 knockdown experiment, the attenuation of dopamine transmission by synapsin III knockdown was less severe in PLCγ1 cKO mice than in control mice. These results indicate that the elevated levels of both VMAT2 and synapsin III in dopamine terminals might contribute to the facilitation of dopamine transmission in PLCγ1 cKO mice. It has been demonstrated that both synapsin I and synapsin II have PLCγ1 binding domains and directly bind to the SH3 domain of PLCγ1^83,84^, while direct or indirect interactions between PLCγ1 and synapsin III are unknown. Our study suggests that PLCγ1 might be a key regulator of dopamine release through potential interaction with synapsin III in dopaminergic axons. The detailed mechanisms by which PLCγ1 interacts with these synaptic molecules need to be investigated in further studies. Additional works are also required to clarify the detailed role of PLCγ1 in the regulation of dopamine release via vesicle trafficking by synapsin III.

Finally, we focused our attention on PIP_2_, which is known to interact with various presynaptic molecules important for synaptic release^86,107^. In our study, we observed that PIP_2_, which is hydrolyzed by PLC, is significantly upregulated in PLCγ1 cKO mice. It was shown that presynaptic molecules such as RIM or Piccolo that can interact with PIP_2_ are present in presynaptic active zone-like sites of dopamine boutons^69^. These results imply that PIP_2_ might be also critical for dopamine release in dopamine boutons. Future studies are needed to reveal the role of PLCγ1 in regulating dopamine release through PIP_2_. Taken together, our findings provide a fundamental understanding of intracellular signaling pathways critical for dopamine release.

### Supplementary information

Supplementary Figures
