# Supplementary material for: PLCγ1 in dopamine neurons critically regulates striatal dopamine release via VMAT2 and synapsin III
Source: Exp Mol Med. 2023 Nov 1;55(11):2357–75. doi: 10.1038/s12276-023-01104-y (PMC10689754; doi:10.1038/s12276-023-01104-y)
Supplement: Supplementary file 1 — Supplementary Figures [file 12276_2023_1104_MOESM1_ESM.pdf]

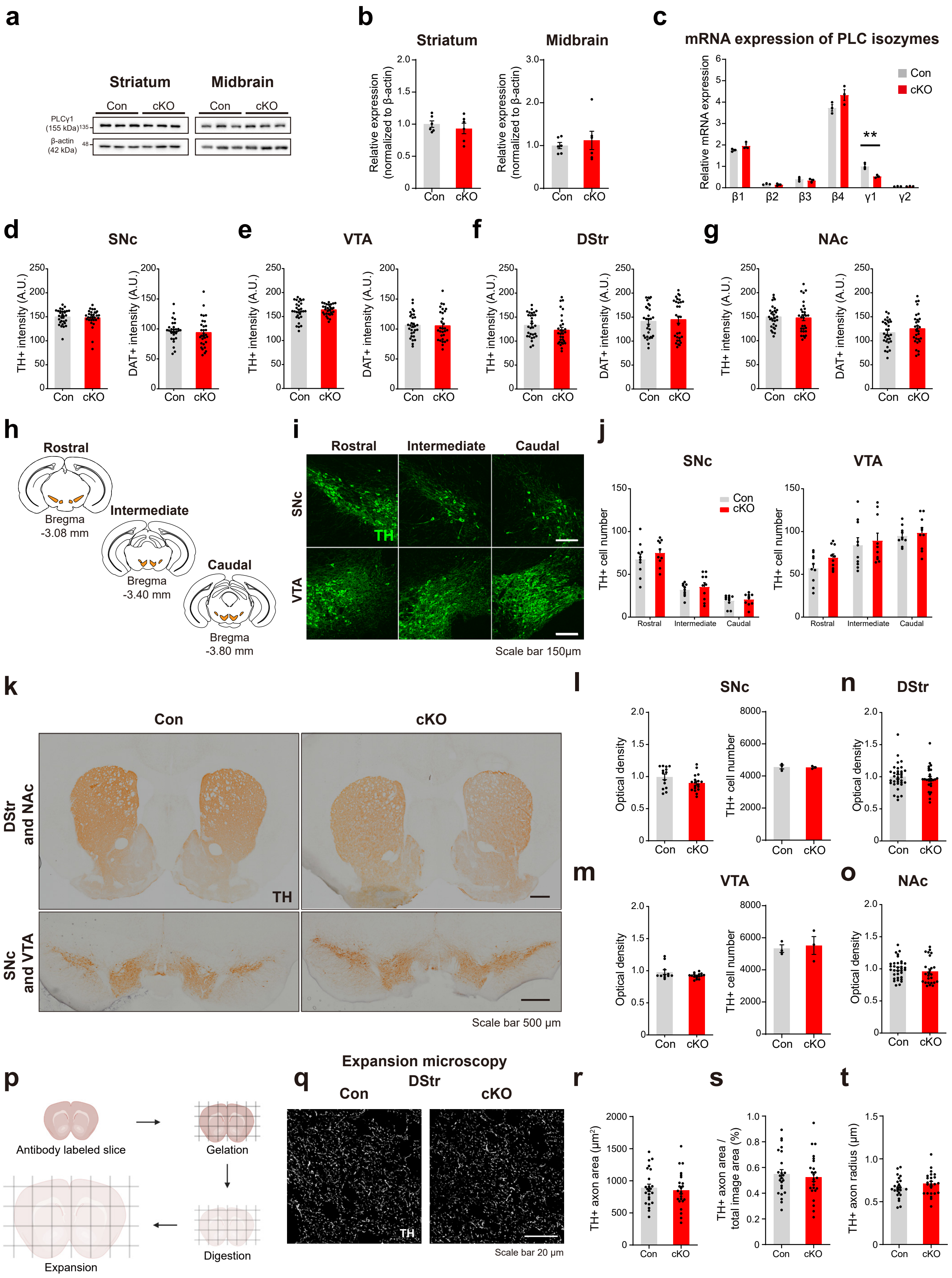
a
Striatum
Midbrain
Con
cKO
PLCγ1 (155 kDa)
135
β-actin (42 kDa)
48
b
Striatum
Midbrain
Relative expression (normalized to β-actin)
c
mRNA expression of PLC isozymes
Relative mRNA expression
β1
β2
β3
β4
γ1
γ2
**
d
SNc
e
VTA
f
DStr
g
NAc
TH+ intensity (A.U.)
DAT+ intensity (A.U.)
h
Rostral
Bregma -3.08 mm
Intermediate
Bregma -3.40 mm
Caudal
Bregma -3.80 mm
i
Rostral
Intermediate
Caudal
SNc
VTA
TH
Scale bar 150μm
j
SNc
VTA
TH+ cell number
k
Con
cKO
DStr and NAc
SNc and VTA
TH
Scale bar 500 μm
l
SNc
m
VTA
n
DStr
o
NAc
Optical density
TH+ cell number
p
Antibody labeled slice
Gelation
Digestion
Expansion
q
Expansion microscopy
DStr
TH
Scale bar 20 μm
r
TH+ axon area (μm²)
s
TH+ axon area / total image area (%)
t
TH+ axon radius (μm)

Supplementary Fig. 1. Expression of TH and DAT is normal in the striatum and midbrain of PLCγ1 cKO mice.

(a) Representative western blot analysis of expression of PLCγ1 in the striatum and midbrain tissue from control and PLCγ1 cKO mice. (b) Quantification of PLCγ1 in the striatum and midbrain by western blot analysis (unpaired two-tailed t-test, n = 6 mice per genotype; Striatum, Con 1 ± 0.05, cKO 0.9326 ± 0.081, p = 0.4991; Midbrain, Con 1 ± 0.072, cKO 1.120 ± 0.214, p = 0.6056). (c) Relative expression of PLC isozymes in dopamine neurons at the RNA level (unpaired two-tailed t-test for each gene, n = 3 mice per genotype, PLCβ1, Con 1.754 ± 0.035, cKO 1.954 ± 0.092, p = 0.1127; PLCβ2, Con 0.171 ± 0.018, cKO 0.144 ± 0.027, p = 0.4616; PLCβ3, Con 0.398 ± 0.058, cKO 0.340 ± 0.047, p = 0.4789; PLCβ4, Con 3.730 ± 0.149, cKO 4.332 ± 0.250, p = 0.1076; PLCγ1, Con 1 ± 0.084, cKO 0.529 ± 0.037, **p = 0.0069; PLCγ2, Con 0.063 ± 0.005, cKO 0.067 ± 0.005, p = 0.6109). (d-g) Fluorescence intensity of TH-positive or DAT-positive areas in the SNc (d), VTA (e), DStr (f), and NAc core (g) (unpaired two-tailed t-test, n = 30 images from 5 mice per genotype; SNc TH+ intensity, Con 149.8 ± 2.893, cKO 147.9 ± 3.432, p = 0.6677; SNc DAT+ intensity, Con 96.34 ± 3.131, cKO 93.81 ± 4.461, p = 0.6445; VTA TH+ intensity, Con 161.4 ± 3.531, cKO 164 ± 2.038, p = 0.5208; VTA DAT+ intensity, Con 106.3 ± 3.608, cKO 105.4 ± 4.501, p = 0.8859; DStr TH+ intensity, Con 132.8 ± 4.539, cKO 122.7 ± 5.681, p = 0.1705; DStr DAT+ intensity, Con 141.2 ± 6.078, cKO 144.7 ± 7.425, p = 0.7170; NAc TH+ intensity, Con 149.6 ± 4.14, cKO 147.5 ± 5.798, p = 0.7753; NAc DAT+ intensity, Con 116.9 ± 4.692, cKO 125.1 ± 6.034, p = 0.2904). (h) Representative coronal sections of the mouse brain, with colored areas indicating SNc and VTA regions (orange). (i-j) Distribution of dopamine neurons at three rostral-to-caudal locations and the quantification of TH-positive dopamine neurons by immunohistochemistry and confocal imaging (SNc, repeated measures two-way ANOVA, n = 10 images at each rostral-to-caudal location from 5 mice per genotype, genotype effect, p = 0.4703; VTA, repeated measures two-way ANOVA, n = 10 images at each rostral-to-caudal location from 5 mice per genotype, genotype effect, p = 0.3614). (k) Representative images of DAB-stained brain slices showing the dopamine neurons in the striatum and midbrain of control and PLCγ1 cKO mice. (l, m) Quantification of optical density of TH immunoreactivity and stereological TH-positive cell counting in the SNc (l) and VTA (m) (unpaired two-tailed t-test, SNc TH optical density, Con 0.99 ± 0.044, n = 14 images from 3 mice, cKO 0.90 ± 0.03, n = 18 images from 3 mice, p = 0.076, SNc TH+ cell number, n = 3 mice per genotype, Con 4562 ± 123.2, cKO 4534 ± 48.04, p = 0.8442, VTA TH optical density, Con 0.98 ± 0.04, n = 11 images from 3 mice, cKO 0.92 ±

0.01, n = 16 images from 3 mice, p = 0.0738, VTA TH+ cell number, n = 3 mice per genotype, Con 5325 ± 236.5, cKO 5519 ± 553.1, p = 0.7631). (n, o) Quantification of optical density of TH immunoreactivity in the DStr (n) and NAc (o) (unpaired two-tailed t-test, DStr, Con 1 ± 0.034, n = 34 images from 5 mice, cKO 0.969 ± 0.038, n = 26 images from 5 mice, p = 0.544; NAc, Con 1 ± 0.003, n = 34 images from 5 mice, cKO 0.9 ± 0.040, n = 25 images from 5 mice, p = 0.3799). (p) Representative schematics of expansion microscopy. (q) Representative expanded images of TH-positive dopamine axons in the DStr. (r-t) Quantification of TH+ axon area (r), TH+ axon density (s), TH+ axon radius (t) in the DStr of control and PLCγ1 cKO mice (unpaired two-tailed t-test, n = 24 images from 4 mice per genotype; TH+ axon area, Con 892.4 ± 55.49 μm^2, cKO 855.3 ± 57.32 μm^2, p = 0.6437; TH+ axon area / total image area, Con 0.549 ± 0.034 %, cKO 0.526 ± 0.035 %, p = 0.6437; TH+ axon radius, Con 0.661 ± 0.024 μm, cKO 0.71 ± 0.027 μm, p = 0.1948).

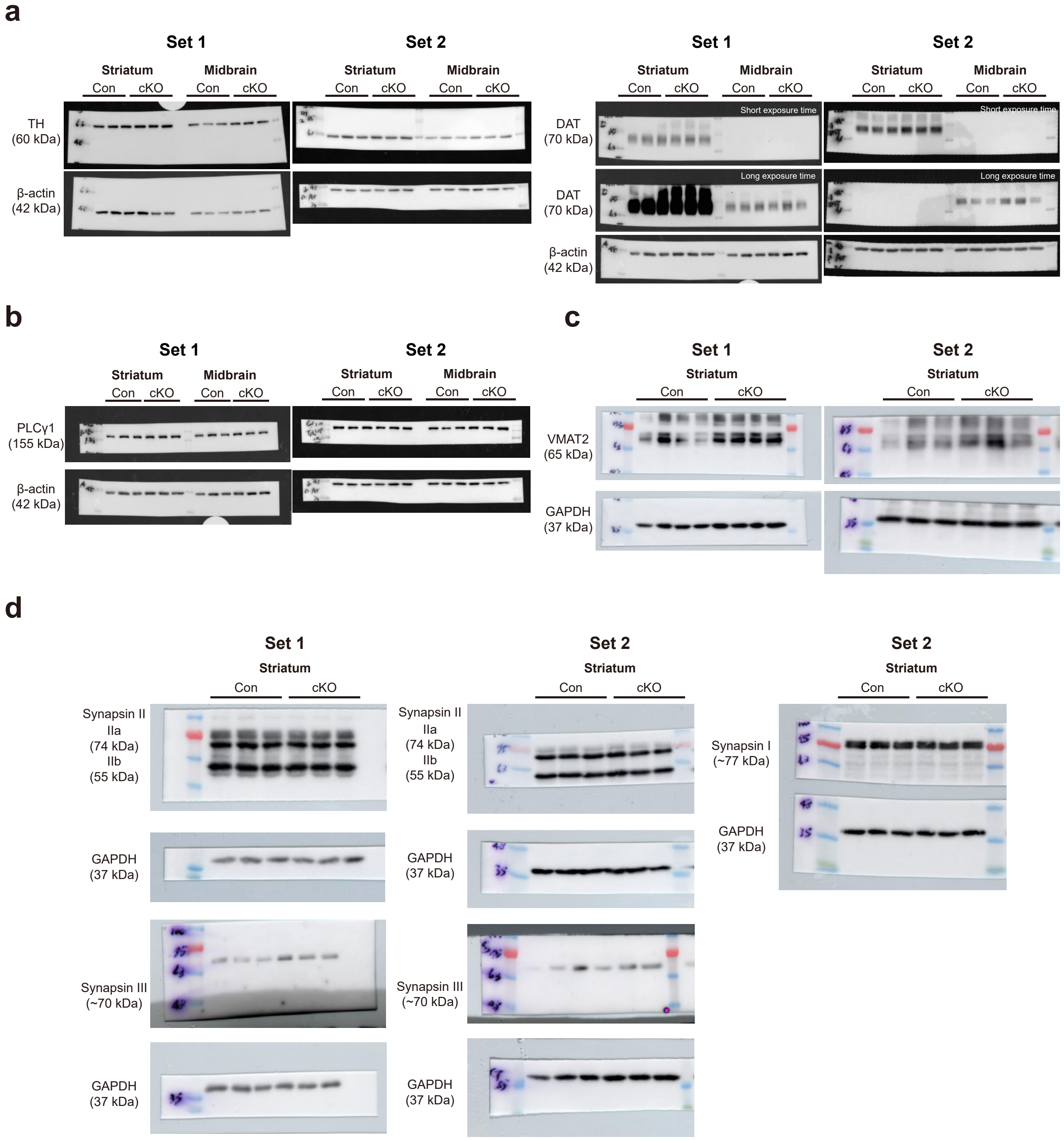
a
Set 1
Striatum
Midbrain
Con
cKO
Set 2
TH
(60 kDa)
β-actin
(42 kDa)
DAT
(70 kDa)
Short exposure time
Long exposure time
b
PLCγ1
(155 kDa)
c
VMAT2
(65 kDa)
GAPDH
(37 kDa)
d
Synapsin II
IIa
(74 kDa)
IIb
(55 kDa)
Synapsin III
(~70 kDa)
Synapsin I
(~77 kDa)

Supplementary Fig. 2. Uncropped western blot images.

(a) Uncropped western blot images of Fig. 1j. (b) Uncropped western blot images of Supplementary Fig. 1a. (c) Uncropped western blot images of Fig. 5a. (d) Uncropped western blot images of Fig. 6a.

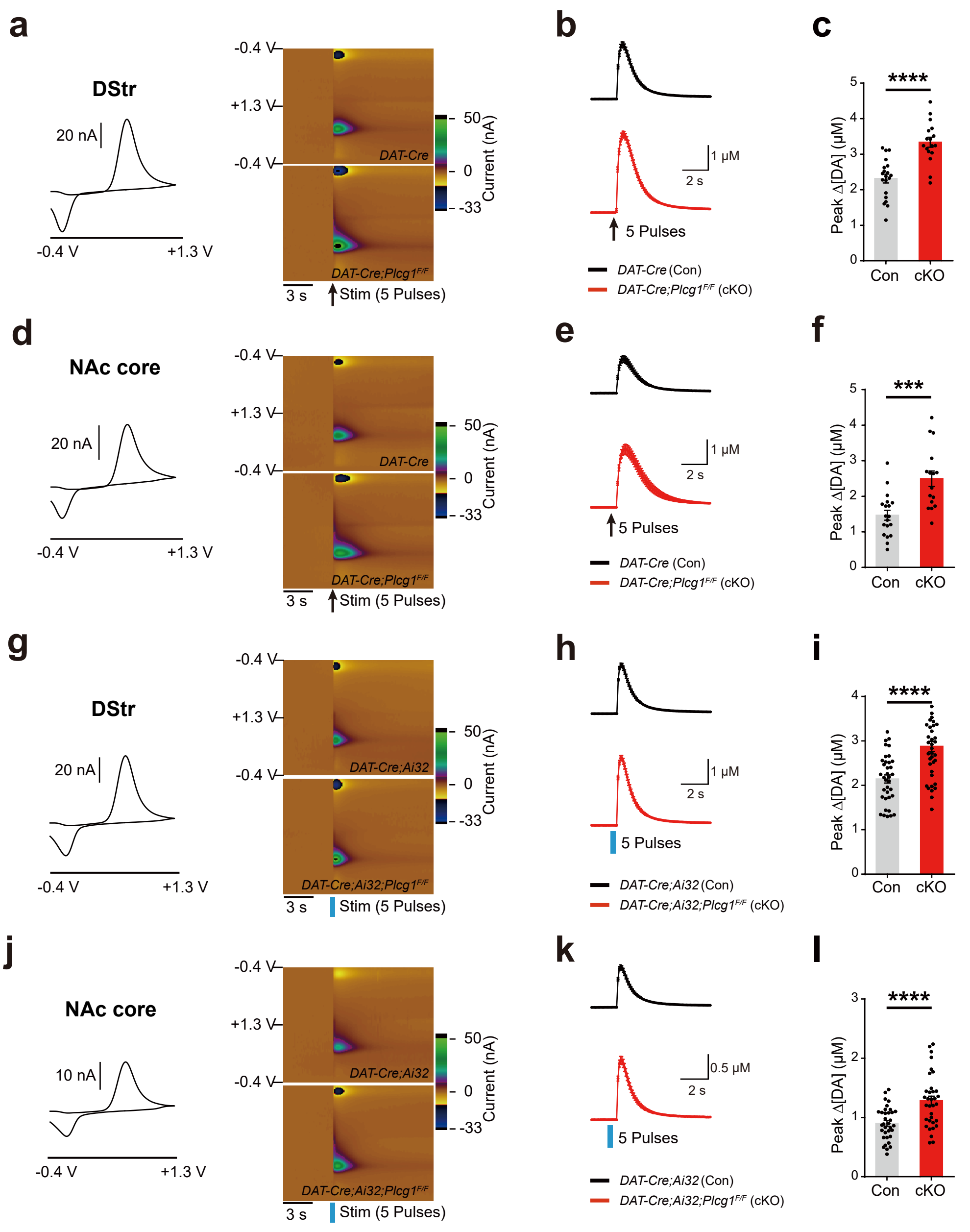
a
DStr
20 nA
-0.4 V
+1.3 V
-0.4 V
+1.3 V
-0.4 V
DAT-Cre
DAT-Cre;Plcg1F/F
3 s
Stim (5 Pulses)
50
0
-33
Current (nA)
b
1 μM
2 s
5 Pulses
DAT-Cre (Con)
DAT-Cre;Plcg1F/F (cKO)
c
****
Peak Δ[DA] (μM)
Con
cKO
d
NAc core
20 nA
e
5 Pulses
f
***
g
DStr
DAT-Cre;Ai32
DAT-Cre;Ai32;Plcg1F/F
h
i
****
j
NAc core
10 nA
k
0.5 μM
l
****
DAT-Cre;Ai32 (Con)
DAT-Cre;Ai32;Plcg1F/F (cKO)

Supplementary Fig. 3. Dopamine release at dopaminergic terminals by 5 pulses stimulation is enhanced by the genetic deletion of PLCγ1.

(a) Representative 2D voltammogram showing oxidation and reduction peaks of dopamine in the DStr (left). Representative 3D color-coded voltammograms from 5 pulses of electrical stimulation in the DStr from control and PLCγ1 cKO mice (right). (b) Summary statistics of dopamine release evoked by 5 pulses of electrical stimulation in the DStr. (c) Quantification of peak dopamine amplitude in the DStr by 5 pulses electrical stimulation (unpaired two-tailed t-test, Con 2.315 ± 0.125 μM, n = 20 slices from 5 mice, cKO 3.337 ± 0.14 μM, n = 17 slices from 4 mice, ****p < 0.0001). (d) Representative 2D voltammogram showing oxidation and reduction peaks of dopamine in the NAc core (left). Representative 3D color-coded voltammograms from 5 pulses of electrical stimulation in the NAc core from control and PLCγ1 cKO mice (right). (e) Summary statistics of dopamine release evoked by 5 pulses of electrical stimulation in the NAc core. (f) Quantification of peak dopamine amplitude in the NAc core by 5 pulses electrical stimulation (unpaired two-tailed t-test, Con 1.466 ± 0.139 μM, n = 19 slices from 5 mice; cKO 2.494 ± 0.218 μM, n = 16 slices from 4 mice, ***p = 0.0003). (g) Representative 2D voltammogram showing oxidation and reduction peaks of dopamine in the DStr (left). Representative 3D color-coded voltammograms from 5 pulses of optogenetic stimulation in the DStr from Control and PLCγ1 cKO mice (right). (h) Summary statistics of dopamine release evoked by 5 pulses of optogenetic stimulation in the DStr. (i) Quantification of peak dopamine amplitude in the DStr by 5 pulses optogenetic stimulation (unpaired two-tailed t-test, Con 2.142 ± 0.092 μM, n = 36 slices from 8 mice, cKO 2.876 ± 0.115 μM, n = 39 slices from 8 mice, ****p < 0.0001). (j) Representative 2D voltammogram showing oxidation and reduction peaks of dopamine in the NAc core (left). Representative 3D color-coded voltammograms from 5 pulses of optogenetic stimulation in the NAc core from control and PLCγ1 cKO mice (right). (k) Summary statistics of dopamine release evoked by 5 pulses of optogenetic stimulation in the NAc core. (l) Quantification of peak dopamine amplitude in the NAc core by 5 pulses optogenetic stimulation (unpaired two-tailed t-test, Con 0.9 ± 0.048 μM, n = 34 slices from 8 mice, cKO 1.282 ± 0.077 μM, n =34 slices from 8 mice, ****p < 0.0001).

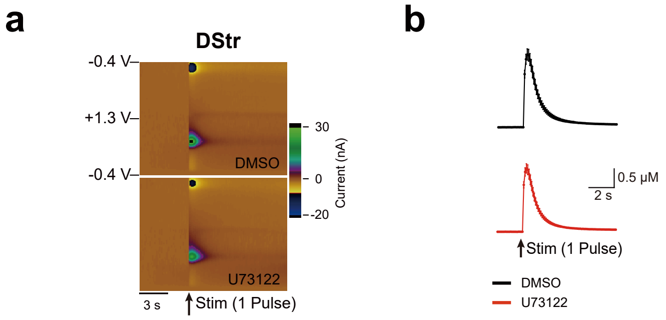

a
DStr
-0.4 V
+1.3 V
-0.4 V
DMSO
U73122
30
0
-20
Current (nA)
3 s
Stim (1 Pulse)
b
0.5 µM
2 s
Stim (1 Pulse)
DMSO
U73122
c
Peak Δ[DA] (µM)
5
4
3
2
1
0
DMSO
U73122

Supplementary Fig. 4. Dopamine release at dopaminergic terminals is not affected by PLC inhibitor.

(a-c) Acute inhibition of PLC does not affect dopamine release in the DStr. (a) Representative 3D color-coded voltammograms evoked by 1 pulse electrical stimulation in the DStr treated with vehicle (DMSO) or U73122 (10 μM). (b) Summary statistics of dopamine release evoked by 1 pulse electrical stimulation in the DStr. (c) Quantification of peak dopamine amplitude in the DStr by 1 pulse electrical stimulation (unpaired two-tailed t-test, $n = 18$ from 6 mice per each group, DMSO 2.005 ± 0.169 μM, U73122 1.687 ± 0.127 μM, $p = 0.1415$).

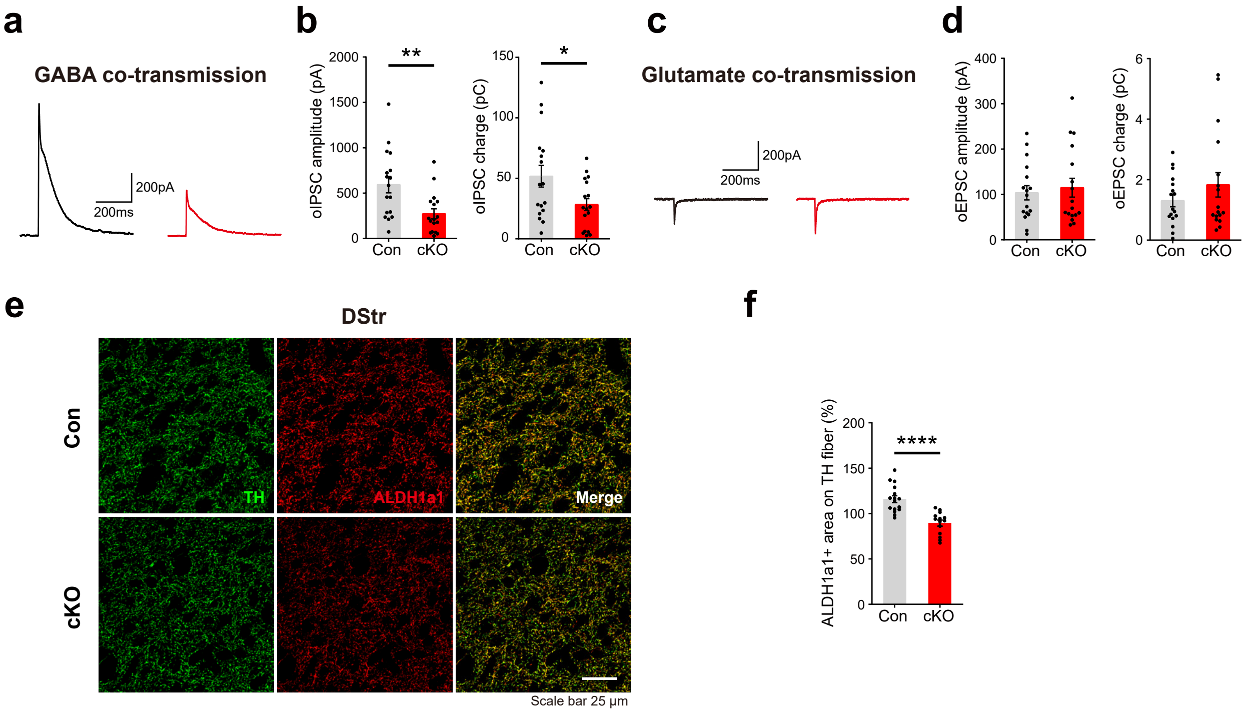

a
GABA co-transmission
200pA
200ms
b
2000
1500
1000
500
0
oIPSC amplitude (pA)
**
Con
cKO
150
100
50
0
oIPSC charge (pC)
*
Con
cKO
c
Glutamate co-transmission
200pA
200ms
d
400
300
200
100
0
oEPSC amplitude (pA)
Con
cKO
6
4
2
0
oEPSC charge (pC)
Con
cKO
e
DStr
Con
cKO
TH
ALDH1a1
Merge
Scale bar 25 μm
f
200
150
100
50
0
ALDH1a1+ area on TH fiber (%)
****
Con
cKO

Supplementary Fig. 5. Downregulation of GABA co-transmission in PLCγ1 cKO mice. (a) Representative recording traces of GABA co-transmission in the DStr of *DAT-Cre;Ai32* and *DAT-Cre;Plcg1$^{F/F}$;Ai32* mice. (b) Summary statistics of oIPSC amplitude (unpaired two-tailed t-test, n = 17 from 4 mice per group; Con 593.7 ± 90.07 pA, cKO 272.8 ± 55.03 pA, **p = 0.0047) and oIPSC charge (unpaired two-tailed t-test, n = 17 from 4 mice per group; Con 51.72 ± 8.992 pC, cKO 28.32 ± 4.940 pC, *p = 0.0294) in the DStr. (c) Representative recording traces of glutamate co-transmission in the DStr of *DAT-Cre;Ai32* and *DAT-Cre;Plcg1$^{F/F}$;Ai32* mice. (d) Summary statistics of oEPSC amplitude (unpaired two-tailed t-test, n = 17 from 4 mice per group; Con 103.7 ± 15.67 pA, cKO 114.8 ± 20.78 pA, p = 0.6731) and oEPSC charge (unpaired two-tailed t-test, n = 17 from 4 mice per group; Con 1.304 ± 0.199 pC, cKO 1.827 ± 0.403 pC, p = 0.2541) in the DStr. (e) Representative confocal images of TH and ALDH1a1 in the DStr of *DAT-Cre* and *DAT-Cre;Plcg1$^{F/F}$* mice. (f) Summary statistics of ALDH1a1 area on TH fibers (unpaired two-tailed t-test, n = 14 images from 4 mice per group; Con 110.3 ± 2.697, cKO 89.46 ± 3.333, ****p < 0.0001).

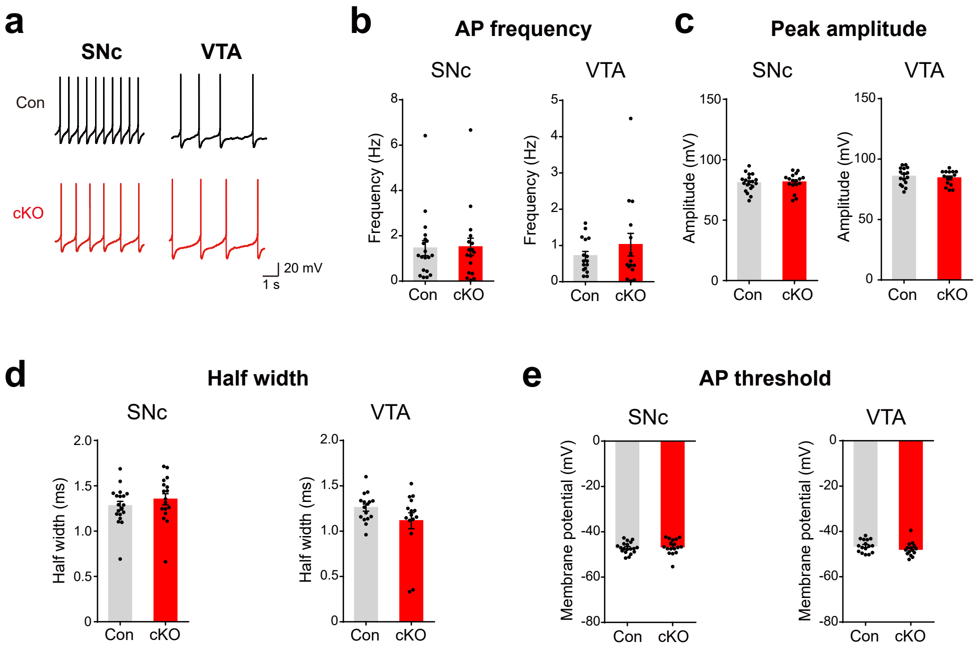
a
SNc
VTA
Con
cKO
20 mV
1 s
b
AP frequency
SNc
VTA
Frequency (Hz)
Con
cKO
c
Peak amplitude
SNc
VTA
Amplitude (mV)
Con
cKO
d
Half width
SNc
VTA
Half width (ms)
Con
cKO
e
AP threshold
SNc
VTA
Membrane potential (mV)
Con
cKO

Supplementary Fig. 6. Intrinsic properties of dopamine neurons are not affected by genetic deletion of PLCγ1.

(a) Representative recording traces of spontaneous action potentials of SNc and VTA dopamine neurons. (b-e) Quantification of action potential frequency (b), peak amplitude (c), half width (d), and action potential threshold (e) (unpaired two-tailed t-test, SNc Con n = 19 cells from 8 mice, SNc cKO n = 17 cells from 7 mice, VTA Con n = 16 cells from 8 mice, VTA cKO n = 15 cells from 7 mice; b, SNc AP frequency, Con 1.457 ± 0.332 Hz, cKO 1.512 ± 0.385 Hz, p = 0.9144, VTA AP frequency, 0.716 ± 0.118 Hz, cKO 1.022 ± 0.311 Hz, p = 0.3524; c, SNc peak amplitude, Con 80.86 ± 1.661 mV, cKO 81.48 ± 1.79 mV, p = 0.7996, VTA peak amplitude, Con 85.69 ± 1.789 mV, cKO 84.38 ± 1.591 mV, p = 0.5902; d, SNc half width, Con 1.28 ± 0.048 ms, cKO 1.351 ± 0.062 ms, p = 0.3656, VTA half width, Con 1.259 ± 0.039 ms, cKO 1.115 ± 0.088 ms, p = 0.1357; e, SNc AP threshold, Con -47.1 ± 0.569 mV, cKO -46.56 ± 0.804 mV, p = 0.5836, VTA AP threshold, Con -46.44 ± 0.691 mV, cKO -47.85 ± 0.796 mV, p = 0.1892).

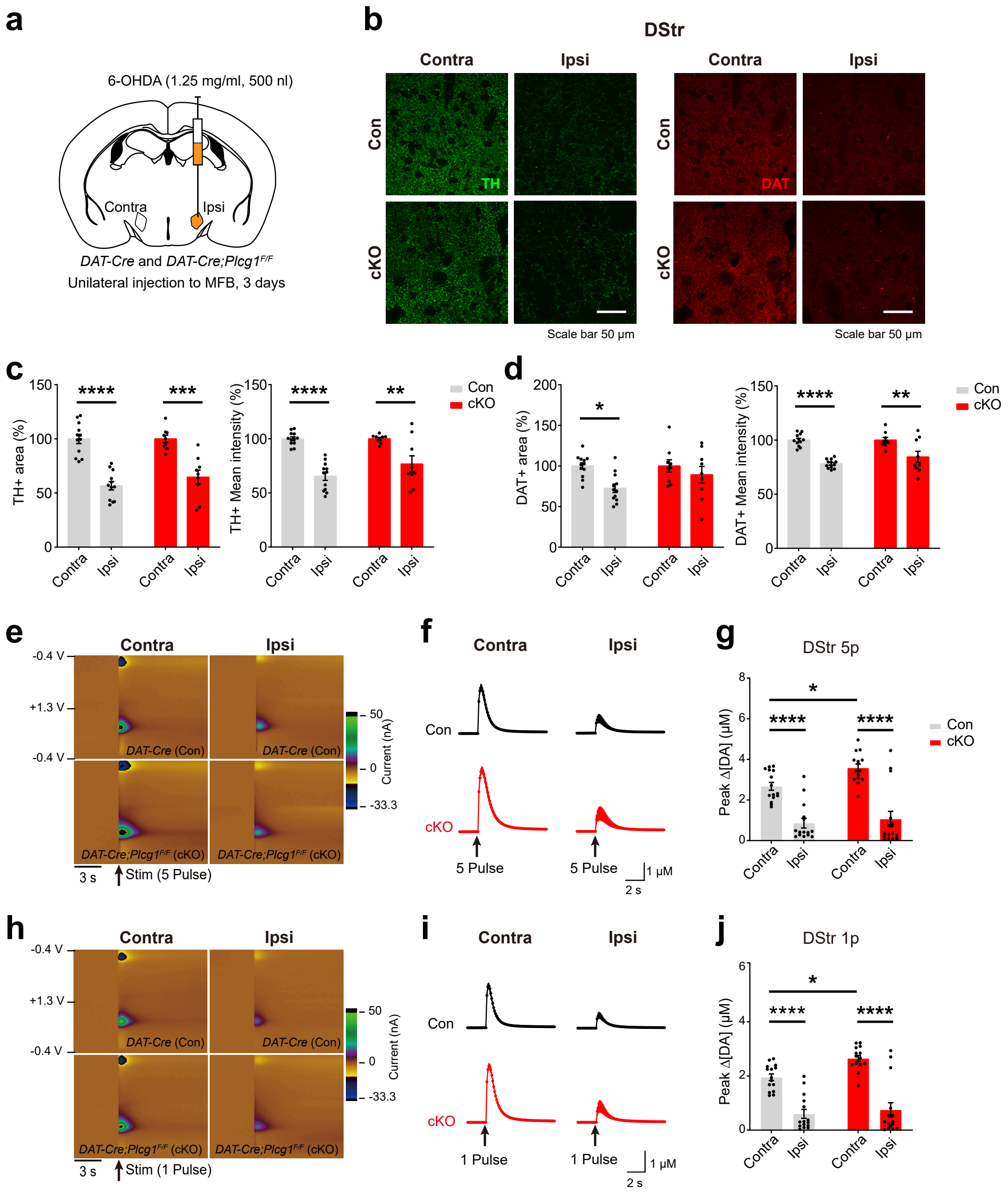
a
6-OHDA (1.25 mg/ml, 500 nl)
Contra
Ipsi
DAT-Cre and DAT-Cre;Plcg1F/F
Unilateral injection to MFB, 3 days
b
DStr
Contra
Ipsi
Con
cKO
TH
DAT
Scale bar 50 μm
c
TH+ area (%)
TH+ Mean intensity (%)
Con
cKO
d
DAT+ area (%)
DAT+ Mean intensity (%)
e
Contra
Ipsi
-0.4 V
+1.3 V
DAT-Cre (Con)
DAT-Cre;Plcg1F/F (cKO)
Current (nA)
3 s
Stim (5 Pulse)
f
5 Pulse
1 μM
2 s
g
DStr 5p
Peak Δ[DA] (μM)
h
Stim (1 Pulse)
i
1 Pulse
j
DStr 1p

Supplementary Fig. 7. Enhanced DA release in PLCγ1 cKO mice does not ameliorate 6-OHDA-induced neurodegeneration.

(a) Schematic illustration describing the injection of 6-OHDA unilaterally into the MFB of *DAT-Cre* and *DAT-Cre;Plcg1*$^{F/F}$ mice. (b) Representative confocal images of TH and DAT in the DStr of each hemisphere of *DAT-Cre* and *DAT-Cre;Plcg1*$^{F/F}$ mice. (c-d) Summary statistics for area and intensity of TH (c) and DAT (d) in the ipsilateral hemisphere compared to the contralateral hemisphere (two-way ANOVA, Con n = 12 slices from 4 mice, cKO n = 9 slices from 3 mice; c, TH area, 6-OHDA effect, ****$p < 0.0001$, Sidak's multiple comparisons test, *DAT-Cre* contra vs. *DAT-Cre* ipsi, ****$p < 0.0001$, *DAT-Cre;Plcg1*$^{F/F}$ contra vs. *DAT-Cre;Plcg1*$^{F/F}$ ipsi, ***$p = 0.0001$; TH mean intensity, 6-OHDA effect, ****$p < 0.0001$, Sidak's multiple comparisons test, *DAT-Cre* contra vs. *DAT-Cre* ipsi, ****$p < 0.0001$, *DAT-Cre;Plcg1*$^{F/F}$ contra vs. *DAT-Cre;Plcg1*$^{F/F}$ ipsi, **$p = 0.0027$; d, DAT area, 6-OHDA effect, *$p = 0.0189$, Sidak's multiple comparisons test, *DAT-Cre* contra vs. *DAT-Cre* ipsi, *$p = 0.0219$; DAT mean intensity, 6-OHDA effect, ****$p < 0.0001$, Sidak's multiple comparisons test, *DAT-Cre* contra vs. *DAT-Cre* ipsi, ****$p < 0.0001$, *DAT-Cre;Plcg1*$^{F/F}$ contra vs. *DAT-Cre;Plcg1*$^{F/F}$ ipsi, **$p = 0.0027$). (e, h) Representative 3D color-coded voltammograms evoked by 5 pulses (e) and 1 pulse (h) electrical stimulation in the DStr of *DAT-Cre* and *DAT-Cre;Plcg1*$^{F/F}$ mice after 6-OHDA injection. (f, i) Summary statistics of dopamine release evoked by 5 pulses (f) and 1 pulse (i) electrical stimulation in the DStr. (g, j) Quantification of peak dopamine amplitude in the DStr by 5 pulses (g) and 1 pulse (j) electrical stimulation (repeated measures two-way ANOVA, Con, cKO n = 15 slices from 5 mice; g, 5 pulses, 6-OHDA effect, ****$p < 0.0001$, Sidak's multiple comparisons test, *DAT-Cre* contra vs. *DAT-Cre* ipsi, ****$p < 0.0001$, *DAT-Cre;Plcg1*$^{F/F}$ contra vs. *DAT-Cre;Plcg1*$^{F/F}$ ipsi, ****$p < 0.0001$, *DAT-Cre* contra vs. *DAT-Cre;Plcg1*$^{F/F}$ contra, *$p = 0.0323$; j, 1 pulse, 6-OHDA effect, ****$p < 0.0001$, genotype effect, *$p = 0.0323$, Sidak's multiple comparisons test, *DAT-Cre* contra vs. *DAT-Cre* ipsi, ****$p < 0.0001$, *DAT-Cre;Plcg1*$^{F/F}$ contra vs. *DAT-Cre;Plcg1*$^{F/F}$ ipsi, ****$p < 0.0001$, *DAT-Cre* contra vs. *DAT-Cre;Plcg1*$^{F/F}$ contra, *$p = 0.0141$).

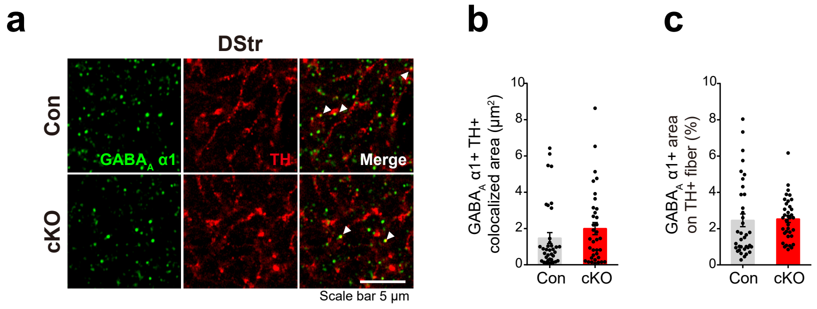

a
DStr
Con
cKO
GABAA α1
TH
Merge
Scale bar 5 μm
b
GABAA α1+ TH+ colocalized area (μm²)
Con
cKO
c
GABAA α1+ area on TH+ fiber (%)
Con
cKO

Supplementary Fig. 8. Expression of $GABA_A$ α1 subunit is comparable between control and PLCγ1 cKO mice in striatal dopaminergic axons.

(a) Representative confocal images of $GABA_A$α1 expression in striatal dopaminergic axons. (b) Quantification of $GABA_A$α1 subunit and TH colocalized area (unpaired two-tailed t-test, n = 36 images from 3 mice per genotype, Con 1.473 ± 0.305 μm^2, cKO 1.997 ± 0.336 μm^2, p = 0.2527). (c) Percentage of $GABA_A$α1-positive area on TH-positive axons (unpaired two-tailed t-test, n = 36 images from 3 mice per genotype, Con 2.459 ± 0.351 %, cKO 2.532 ± 0.196 %, p = 0.8573).

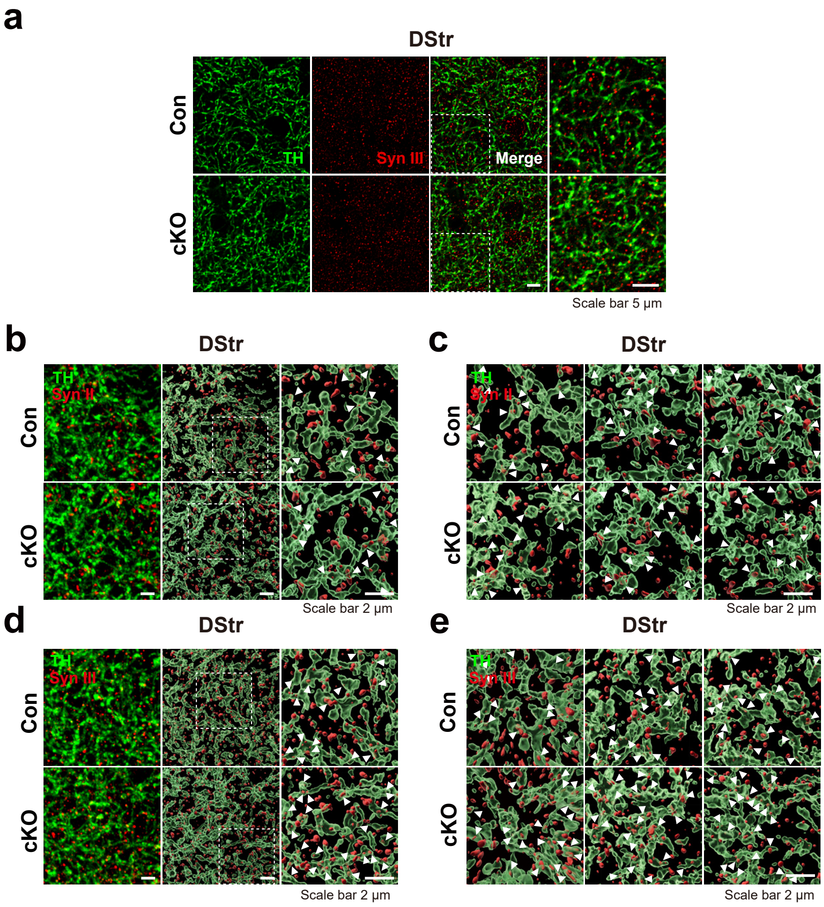
a
DStr
Con
cKO
TH
Syn III
Merge
Scale bar 5 μm
b
DStr
TH
Syn II
Con
cKO
Scale bar 2 μm
c
DStr
TH
Syn II
Con
cKO
Scale bar 2 μm
d
DStr
TH
Syn III
Con
cKO
Scale bar 2 μm
e
DStr
TH
Syn III
Con
cKO
Scale bar 2 μm

Supplementary Fig. 9. Expression of synapsins in striatal dopaminergic axons.

(a) Representative confocal images of synapsin III expression in striatal dopaminergic axons. (b) Representative z stack confocal images of TH and synapsin II showing the expression of synapsin II in striatal dopaminergic axons (left), IMARIS 3D projections of IHC staining (middle), enlarged 3D representation of the selected area (right, white arrowhead: TH and synapsin II colocalization). (c) Representative 3D reconstruction images of TH and synapsin II using IMARIS software (white arrowhead: TH and synapsin II colocalization). (d) Representative z stack confocal images of TH and synapsin III showing the expression of synapsin III in striatal dopaminergic axons (left), IMARIS 3D projections of IHC staining (middle), enlarged 3D representation of the selected area (right, white arrowhead: TH and synapsin III colocalization). (e) Representative 3D reconstruction images of TH and synapsin III using IMARIS software (white arrowhead: TH and synapsin III colocalization).

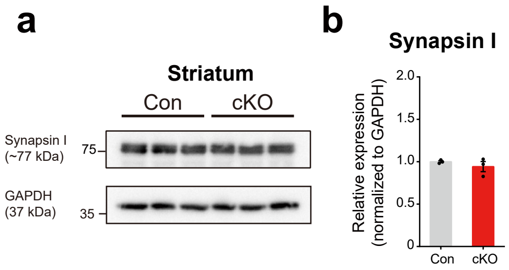
a
Striatum
Con
cKO
Synapsin I
(~77 kDa)
75
GAPDH
(37 kDa)
35
b
Synapsin I
Relative expression
(normalized to GAPDH)
2.0
1.5
1.0
0.5
0
Con
cKO

Supplementary Fig. 10. Synapsin I expression is not altered in PLCγ1 cKO mice.

(a) Representative western blot images of synapsin I expression in striatal synaptosomes in control and PLCγ1 cKO mice. (b) Quantification of synapsin I expression in striatal synaptosomes (unpaired two-tailed t-test, n = 3 mice per genotype, Con 1 ± 0.011, cKO 0.9423 ± 0.06, p = 0.4007).

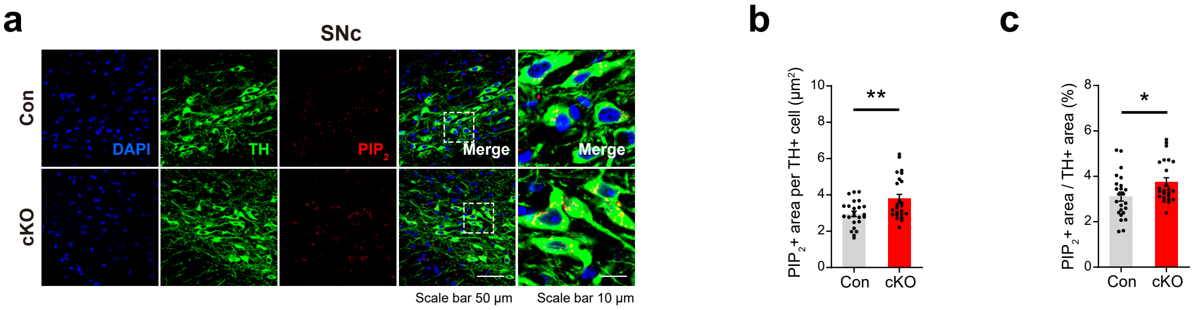

a
SNc
Con
cKO
DAPI
TH
PIP2
Merge
Merge
Scale bar 50 μm
Scale bar 10 μm
b
PIP2+ area per TH+ cell (μm2)
**
Con
cKO
c
PIP2+ area / TH+ area (%)
*
Con
cKO

Supplementary Fig. 11. Expression of PIP_2 in SNc dopamine neurons.

(a) Representative confocal images of PIP_2 levels in the SNc dopamine neurons of control and PLCγ1 cKO mice. (b-c) Quantification of PIP_2-positive area per dopamine neuron (b) and TH-positive area (c) (unpaired two-tailed t-test, n = 24 images from 4 mice per genotype, PIP_2-positive area per dopamine neuron, Con 2.951 ± 0.148 μm^2, cKO 3.790 ± 0.240 μm^2, **p = 0.0047, PIP_2-positive area per TH-positive area, Con 3.108 ± 0.197 %, cKO 3.749 ± 0.189 %, *p = 0.0236).
